# X-ray tomography of cryopreserved human prostate cancer cells: mitochondrial targeting by an organoiridium photosensitiser

**DOI:** 10.1007/s00775-020-01761-8

**Published:** 2020-03-02

**Authors:** Elizabeth M. Bolitho, Carlos Sanchez-Cano, Huaiyi Huang, Ian Hands-Portman, Matthew Spink, Paul D. Quinn, Maria Harkiolaki, Peter J. Sadler

**Affiliations:** 1 Department of Chemistry, University of Warwick, Gibbet Hill Road, Coventry, CV4 7AL UK; 2 Diamond House, Harwell Science and Innovation Campus, Fermi Ave, Didcot, OX11 0DE UK; 3 Center for Cooperative Research in Biomaterials (CIC biomaGUNE), Basque Research and Technology Alliance (BRTA), Paseo Miramon 182, 20014 Donostia-San Sebastián, Spain; 4 School of Life Sciences, University of Warwick, Gibbet Hill Campus, Coventry, CV4 7AL UK

**Keywords:** Photodynamic therapy, Iridium complexes, Phosphorescence, Anticancer metallodrugs, Cryo-soft X-ray tomography, X-ray microscopy

## Abstract

**Abstract:**

The organoiridium complex Ir[(C,N)_2_(O,O)] (**1**) where C, N = 1-phenylisoquinoline and O,O = 2,2,6,6-tetramethyl-3,5-heptanedionate is a promising photosensitiser for Photo-Dynamic Therapy (PDT). **1** is not toxic to cells in the dark. However, irradiation of the compound with one-photon blue or two-photon red light generates high levels of singlet oxygen (^1^O_2_) (in Zhang et al. Angew Chem Int Ed Engl 56 (47):14898-14902 10.1002/anie.201709082,2017), both within cell monolayers and in tumour models. Moreover, photo-excited **1** oxidises key proteins, causing metabolic alterations in cancer cells with potent antiproliferative activity. Here, the tomograms obtained by cryo-Soft X-ray Tomography (cryo-SXT) of human PC3 prostate cancer cells treated with **1**, irradiated with blue light, and cryopreserved to maintain them in their native state, reveal that irradiation causes extensive and specific alterations to mitochondria, but not other cellular components. Such new insights into the effect of ^1^O_2_ generation during PDT using iridium photosensitisers on cells contribute to a detailed understanding of their cellular mode of action.

**Graphic abstract:**

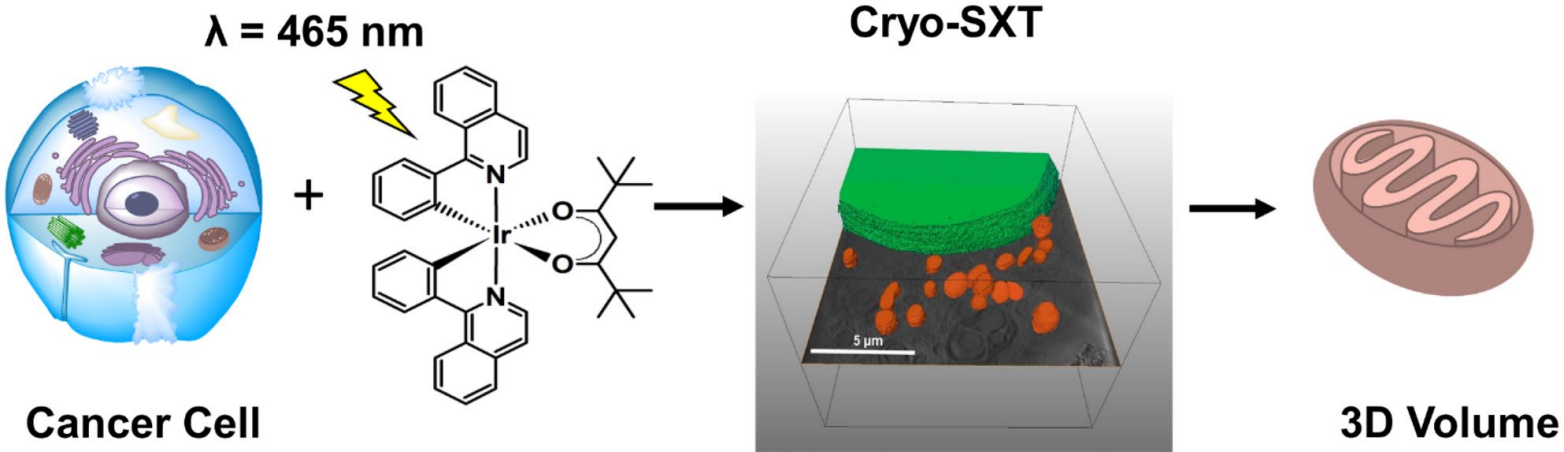

**Electronic supplementary material:**

The online version of this article (10.1007/s00775-020-01761-8) contains supplementary material, which is available to authorized users.

## Introduction

Photodynamic Therapy (PDT) is a non-invasive therapeutic approach used to treat surface cancers. It uses spatially directed visible light (*λ* = 500–800 nm) to irradiate a photosensitiser prodrug, which is non-toxic in the dark [2–7]. This generates reactive oxygen species (ROS)—particularly singlet oxygen (^1^O_2_)—in a highly confined manner within the irradiated area, subsequently inducing apoptosis locally in the tumour [8–10]. As such, PDT can help to reduce unwanted side effects normally experienced by patients treated with other chemotherapeutic agents [11].

**Fig. 1:**
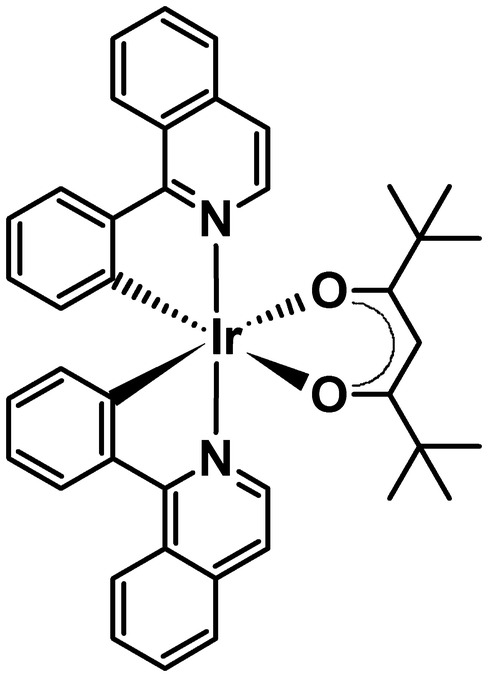
Structure of [Ir(C,N)_2_(O,O)] [1] (where C,N = 1-phenylisoquinoline and O,O = 2,2,6,6-tetramethyl-3,5-heptanedionate) [1]

Cyclometallated organoiridium(III) octahedral complexes are promising photosensitisers for PDT [12–22]. They possess high photostability and long luminescence lifetimes, together with an ability to permeate cells [23], and generate toxic ROS upon irradiation. In particular, the organoiridium complex Ir[(C,N)_2_(O,O)] (**1)** (C, N = 1-phenylisoquinoline and O,O = 2,2,6,6-tetramethyl-3,5-heptanedionate, Fig. 1) and its analogues have shown outstanding biological properties. Complex **1** has a long phosphorescent lifetime (*λ*_ex_ = 458 nm; *λ*_p_ = 620 nm; *τ*_p_ = 59 ns) in the presence of oxygen [1], and exerts potent antiproliferative activity after one- or two-photon activation with low doses of visible light [1]. This generates high amounts of highly toxic singlet oxygen (^1^O_2_) and causes profound oxidative damage to proteins important for cell metabolism (i.e. histidine residues on aldose reductase and heat-shock protein-70, HsP-70) [1].

Notably, the oxidative stress triggered by irradiating cells treated with **1** increases the expression of enzymes of the glycolytic pathway [1], apparently suggesting a higher dependency of the cells on the glucose metabolism. Thus, **1** might cause alterations in mitochondrial activity leading to depletion of the metabolism involving these organelles. Structural information at a sub-cellular level on the effects caused by the irradiation of cells treated with **1** can make an important contribution towards our understanding of the mechanism of anticancer activity of this family of PDT photosensitisers. Furthermore, mitochondria have been proposed previously as major target sites for a variety of other iridium photosensitisers [16, 18, 19, 24], so there is wider interest in developing methods which allow the direct investigation of the effects of photo-irradiation of such complexes on cellular organelles.

Cryo-Soft X-ray Tomography (cryo-SXT) is an X-ray-based technique which operates at the ‘water-window’ (spectral region between the K edges of carbon and oxygen atoms, ca 280–530 eV, where structured carbon-rich biological matter absorbs light significantly more than the surrounding oxygen-rich medium) [25]. This generates natural contrast and allows imaging of the inner structure of cryopreserved whole cells without the need for further contrast through the use of additional chemical staining [26]. Cryo-SXT makes use of samples that have been vitrified, to immobilise and cryo-preserve cell structure, and therefore delivers 3D imaging of hydrated biological samples (cells and thin tissue slices) under near-native conditions at high resolution (in the tens of nanometre range) [27, 28]. This provides detailed in situ structural information on the distribution and morphology of cell organelles [29, 30]. Hence, this technique has the potential to provide remarkable insights into the changes induced upon the exposure of cells to activated metallodrug candidates [31]. Here, we have used cryo-SXT at the biological cryo-imaging beamline B24 of the UK synchrotron, Diamond Light Source, to probe the effects of the photoactivation of **1** on the cellular organelles of human PC3 prostate cancer cells.[32]

## Materials and methods

### Materials

200 mesh gold TEM F1finder grids with an R2/2 quantifoil carbon substrate were purchased from Quantifoil Micro Tools GmbH (Germany). Gold nanoparticle fiducials (AuNP) of 250 nm diameter were purchased from BBI Solutions (UK). MitoTracker Red FM 580 (excitation/emission = 581/644 nm) was purchased from Fischer Scientific (UK). Molecular-grade DMSO (Bioultra, GC ≥ 99.5%) was purchased from Sigma Aldrich (UK). RPMI-1640 (non-phenol red) culture medium supplemented with penicilin/streptomycin, L-glutamine and Fetal bovine serum (details in SI) was supplied by the Media Preparation Service in the School of Life Sciences (University of Warwick). PC3 human prostate adenocarcinoma cells (Catalogue Number 90112714) were purchased from the European Collection of Cell Cultures (ECACC). Cells were tested every 6 months to confirm mycoplasma-free status.

### Synthesis of 1

[Ir(C,N)_2_(O,O)] **(1)** was synthesised and fully characterised following previously reported methods [1].

### Growth of PC3 cells on quantifoil TEM Grids

Quantifoil F1 TEM grids (3.05 mm diameter) were deposited in 24-well tissue culture plates (Greiner Bio-One, UK; 1 grid/well) and irradiated with UV-light for 20 min to sterilise. PC3 human prostate cancer cells were seeded on the grids (1 × 10^5^ cells/mL; 2 mL/well) and incubated for 24 h (37 °C, 5% CO_2_) in RPMI-1640 cell culture medium (supplemented with 10% fetal bovine serum, 1% penicillin/streptomycin and 1% L-glutamine (2 mM)). After 24 h, the medium was removed, and cells were treated with 0 or 1 µM of **1** (1/6th of the half-maximal inhibitory concentration, IC_50_; prepared in 5% DMSO, 95% non-phenol red RPMI-1640) for 2 h in the dark. The supernatant was removed after incubation and replaced with fresh RPMI-1640 medium. Cells were then incubated for 10 min at room temperature under either (a) dark conditions (no light exposure) or (b) irradiation with blue light (465 nm, 4.8 mW/cm^2^), followed by further incubation for 24 h in the dark (37 °C, 5% CO_2_). After this, Mitotracker Red FM (*λ*_ex/em_ = 581/644 nm) was added to a final concentration of 1 nM to all samples for 15 min. The supernatant was thereafter removed and cells were washed with PBS (× 2), gold nanoparticle fiducials added (ca. 3.6 × 10^7^ particles in 10 μL), blotted with filter paper (1 s) to remove the excess liquid, and plunge-frozen in liquid propane:ethane mixture (cooled in liquid nitrogen). The resulting vitrified samples were stored under liquid nitrogen vapour phase for further analysis and imaging.

### Cryo-SXT experiment

All cryo-SXT imaging was performed on an UltraXRM-S220C (Carl Zeiss X-ray Microscopy, Inc) at beamline B24 at Diamond Light Source (Didcot, UK).

First, fluorescence imaging of MitoTracker Red (*λ*_ex/em_ = 581/644 nm) distribution was used to locate mitochondria on the cryo-preserved grids using optical microscopy (50X) on an Axioimager M2 (Zeiss) coupled to a CMS196M LED cryo-correlative stage (Linkam Scientific Instruments Ltd). This allowed identification of suitable cell candidates for cryo-SXT analysis (vitrified whole cells on intact grid surfaces without ice contamination). Mapped samples (brightfield and fluorescence imaging) were then transferred to the X-ray microscope, under cryogenic conditions (vitrified status was maintained throughout sample transfer and data collection). Initial 2D inspection of whole grid squares was achieved by forming image mosaics of consecutive regions in areas of interest (using single field of view size of 16 × 16 μm^2^) collected with brief exposure to soft X-rays (0.5 s, 500 eV) and detected in continuous mode using a CCD camera (Pixis, XO 1024B; Princeton Scientific). Selected areas were placed on the microscope stage’s centre of rotation through tracking gold nanoparticle fiducials or other dense cell features (i.e. lipid droplets) between tilt angles − 30 and + 30° and imaging focus to the desired depth in the cells was adjusted by moving the 40 nm zone plate objective. Tilt series of images of areas of interest were acquired using a − 65° to + 65° sample tilt (total rotation: 130°) at 0.5º rotation steps. Depending on sample thickness, different exposure times were required to collect tomograms from PC3 cancer cells: (i) Untreated control (Dark) = 6 s (at high tilt angles) and 3 s (at low tilt, 0°); (ii) Untreated control (465 nm) = 2 s (high tilt) and 1 s (low tilt); (iii) **1** (Dark) = 4 s (high tilt) and 2 s (low tilt); (iv) **1** (465 nm) = 8 s (high tilt) and 4 s (low tilt). This relates to the attenuation of X-rays due to variable thickness of samples (influenced by cell density and size, as well as solution content adjacent to target areas).

### Tomogram reconstruction

Raw tilt series data (.tiff files) were reconstructed using IMOD software (which contained the eTomo and 3dmod tools) [33, 34]. The files were converted from tiff to MRC format and the following reconstruction settings were used: pixel size = 15.8 nm, fiducial diameter = 250 nm, image rotation = 0°, starting angle = − 65*°*, increment step = 0.5*°*. The raw image stack was viewed before creating the command scripts to identify views to exclude in the tomogram reconstruction. Then, an X-ray model and a ‘fixed stack’ (which creates a secondary stack with the X-rays removed) were generated. Cross-correlation of still images was calculated with a high-frequency cut off radius of 0.1. Coarse-aligned stacks and fiducial models were generated automatically for each tomogram; fiducials incorrectly assigned by the software were removed and any unassigned fiducials were added manually for all angles and views. Tilt series were then fine-aligned relative to adjacent views with global variation settings of no rotation and fixed magnification of 1. The requested tomogram depth was 1000 (binning = 3). Boundary models were generated and cropped to contain cellular information only. The final aligned stacks were then generated from the calculated *z*-axis and pitch angles using linear interpolation, producing tomograms with no positional drifts. Reconstructed tomograms were generated in 3dmod [33, 34] and post-processed to remove unwanted information (noise or background), saved as reconstruction files ready for analysis by SuRVoS [35].

### Segmentation

The reconstructed tomograms (.rec) were processed using SuRVoS [35]. The region of interest (ROI) was selected appropriately for each tomogram by deleting specific angle views in which thick ice or gridlines obstructed the view. A Gaussian filter feature channel was used and supervoxels (SP = 8 × 8 × 8) were generated in Gaussian mode. Mitochondria annotations were added by manually selecting mitochondrial regions in the supervoxels setting; the mitochondria were located automatically by the software and corrected manually every five to ten views. For each tomogram, 15–30 mitochondria were selected and analysed. From this, the average mitochondrial volume (µm^3^) was calculated for each PC3 cell tomogram under the specified conditions. Additionally, the nucleus was segmented. The exported files were visualised in Amira (Avizo 2016) [36] software using the volume-rendering and ortho slice settings to generate images and videos of each reconstructed and segmented tomograms.

## Results and discussion

Prostate cancer is the most common cancer in men (with over 1 million reported cases in 2013) [37, 38] and a strong candidate for treatment using PDT [32, 39]. We probed the effect of the photoactivation of **1** on the mitochondria, and other organelles, of PC3 human prostate cancer cells. This complex was used as a racemic mixture, and although the enantiomers may be recognised differently by transport systems and other biomolecules, chirality is not expected to affect the photosensitisation properties of **1** such as the generation of singlet oxygen. As expected, the complex was not toxic to PC3 cells under dark conditions (IC_50_ > 50 µM), but showed potent antiproliferative activity (IC_50_: 6 ± 1 µM) when cells were treated with **1** for 2 h followed by blue light irradiation (10 min *λ*_irr_: 465 nm, 4.8 mW/cm^2^; cells left to recover for 46 h before assay). Also, irradiation of untreated cells with blue light under the same conditions did not have any effect on their proliferation. Using this information, we generated vitrified samples of PC3 cells treated with **1** (1 μM; 1/6th IC_50_) and exposed to dark (non-toxic) or irradiated (blue light) conditions, and stained with MitoTracker Red for cryo-SXT.

Suitable cell candidates for X-ray analysis were initially selected by mapping the grids at 77 K (following bright-field, and mitotracker red using the YFP filter; Fig. S1–S4) on a cryogenic optical microscope. Faint green fluorescence was observed in cells treated with **1** when irradiated with blue light (465 nm) and using a microscopy filter designed for GFP (*λ*_ex/em_ = 488/510 nm; Fig. S4). This signal can be attributed to the tail of the phosphorescence emission band of the complex, as **1** shows deep-red emission upon irradiation with light of a similar wavelength (*λ*_ex_ = 458 nm; *λ*_p_ = 620 nm) [1]. Samples and controls were then loaded into the autosampler chamber at B24 and maintained in cryogenic conditions throughout all data collection.

**Fig. 2:**
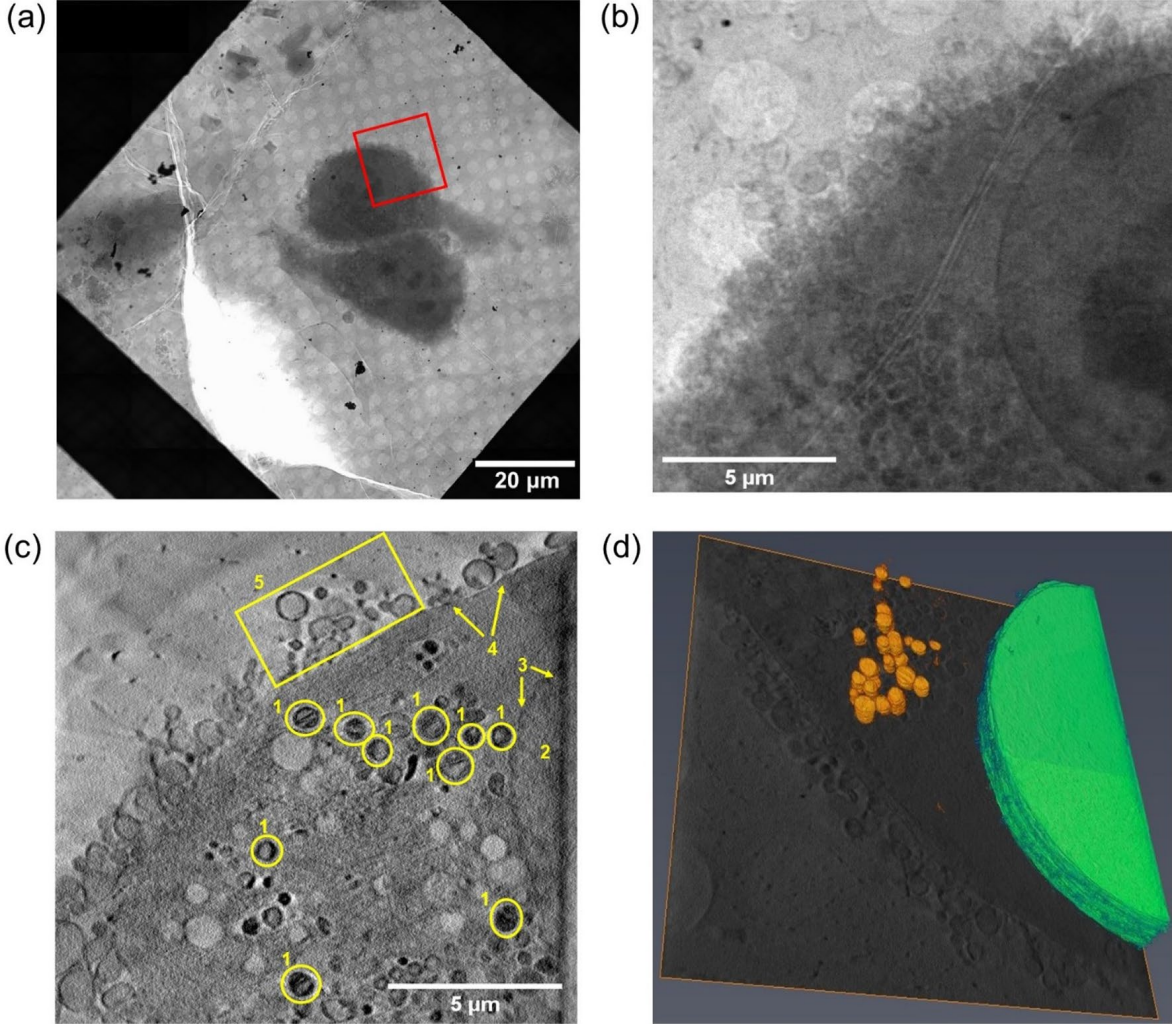
Cryo-SXT images of cryogenically-fixed PC3 prostate cancer cells grown on carbon–gold TEM grids (24 h) and treated with 1 µM of **1** for 2 h, followed by 10 min irradiation with blue light (*λ* = 465 nm) and 24 h recovery in complex-free medium: **a** X-ray mosaic image of a 100 × 100 μm^2^ area of the grid; **b** X-ray projection image collected from a 16 × 16 μm^2^ area of interest (marked as a red square) in **a** after exposure to soft x-rays (8 s, 500 eV); **c** 2D projection of the reconstructed tomogram obtained from the same area (in **b**); showing 1 = mitochondria, 2 = nucleus, 3 = nuclear membrane, 4 = plasma membrane, 5 = vesicles; **d** 3D segmented tomogram analysed in SuRVoS and visualised in Amira; showing mitochondria (orange) and nucleus (green). Images were generated using ImageJ (**a**) [40], IMOD (**b**, **c**) [33, 34] and SuRVoS and Amira imaging softwares (**d**) [34, 36]

We used soft X-rays (500 eV) focused to a 40 × 40 nm^2^ spot size to collect fast mosaic maps of the grid squares. This allowed localisation of PC3 cells previously identified using fluorescence microscopy (Fig. 2a). Remarkably, samples treated with the Ir complex **1** showed increased X-ray absorption, requiring longer exposure times at high tilt angles even when control samples were covered by thicker ice layers (i.e. 6 s for untreated control cell in dark compared to 8 s for cells treated with **1** upon blue light irradiation). The need for this increase is likely due to the concentrations of highly electron-dense iridium complex within the cells. Nevertheless, it was possible to collect clear X-ray projection images from all samples. These images permitted clear observation of the cellular and nuclear membranes, the nucleus, and the nucleolus. Moreover, they also allowed identification of a large number of cytoplasmic vesicles and organelles such as mitochondria (Fig. 2b, Fig. S1–S4).

**Fig. 3:**
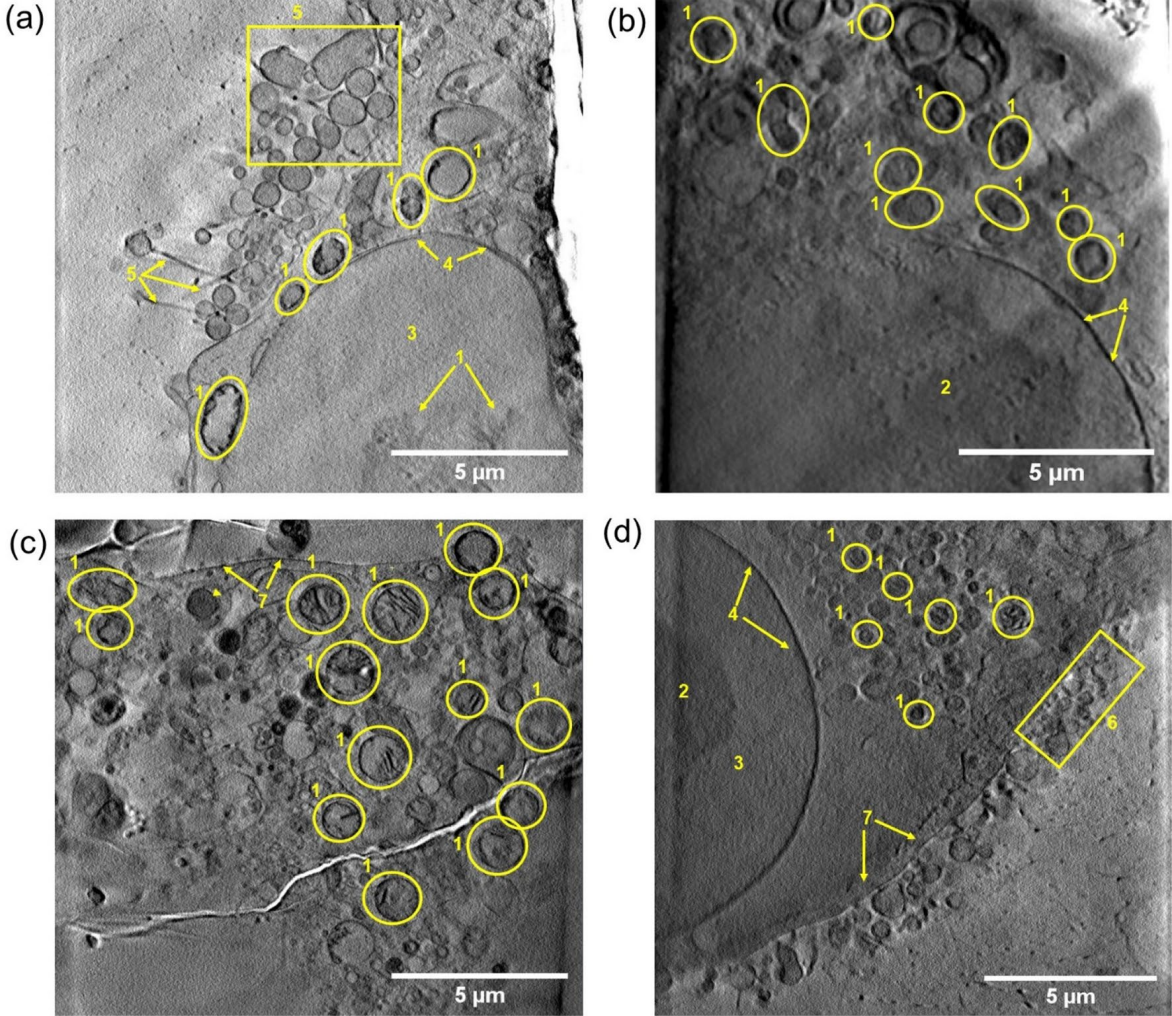
2D projections of reconstructed X-ray tomograms (16 × 16 μm^2^) of cryogenically fixed PC3 human prostate carcinoma cells grown on Quantifoil TEM grids showing changes in cellular and organelle morphology upon treatment with **1**, followed by irradiation with blue light. **a** Untreated cells in dark conditions (not irradiated with blue light; 465 nm). **b** Untreated cells exposed to blue light (10 min, 465 nm, 4.8 mW/cm^2^). **c** Cells treated with **1 (**1 μM) under dark conditions (not irradiated with blue light; 465 nm). **d** Cells treated with **1 (**1 μM) for 2 h, followed by exposure to blue light (10 min, 465 nm, 4.8 mw/cm^2^). Cellular features shown: 1 = mitochondria, 2 = nucleolus, 3 = nucleus, 4 = nuclear membrane, 5 = features of lamellipodium, 6 = spherical vesicles, 7 = plasma membrane. Images were generated using IMOD software [33, 34]

Cells were further studied using cryo-SXT. A total of ten X-ray tomograms (T1-T10; Fig. 2c, Fig. S5–S14; Videos SV1–SV10) were collected from 16 × 16 µm^2^ regions of interest in cells untreated or treated with 1 μM of **1**, either kept in the dark, or irradiated with blue light (10 min *λ*_irr_: 465 nm, 4.8 mW/cm^2^). As expected, both irradiated and non-irradiated untreated PC3 cells possessed undamaged cellular membranes and nucleus, and showed large similarities in their cellular and organelle morphology (Fig. 3a, b, S5–S7; Videos SV1–SV3). In particular, mitochondria, distinguished from other vesicular organelles by the presence of cristae [41], were ellipsoid in shape and located close to the nucleus. Additionally, *lamelipodia* (flat cellular protrusions) [42] associated with a number of spherical vesicular structures were visible near the plasma membrane of the untreated cells (Fig. 3a, S5–S6; Videos SV1–SV2). Such accumulation may correspond to the shedding of membrane vesicles (i.e. exosomes and oncosomes) [43], a feature reported previously for PC3 cells [44–47]. Likewise, the morphology of cells treated with 1 μM of **1** but not irradiated was closely related to that observed for untreated controls (both irradiated and non-irradiated). Tomograms of the latter showed well-structured nuclei, undamaged plasma membranes with vesicle-shedding and *lamellopodia*, and apparently healthy mitochondria with well-defined cristae (Fig. 3c, Fig. S8–S11; Videos SV4–SV7). Therefore, these results correlated with the lack of toxicity of **1** when cells were not irradiated, confirming that neither blue light nor treatment with complex **1** under dark conditions resulted in mitochondrial alterations.

**Fig. 4:**
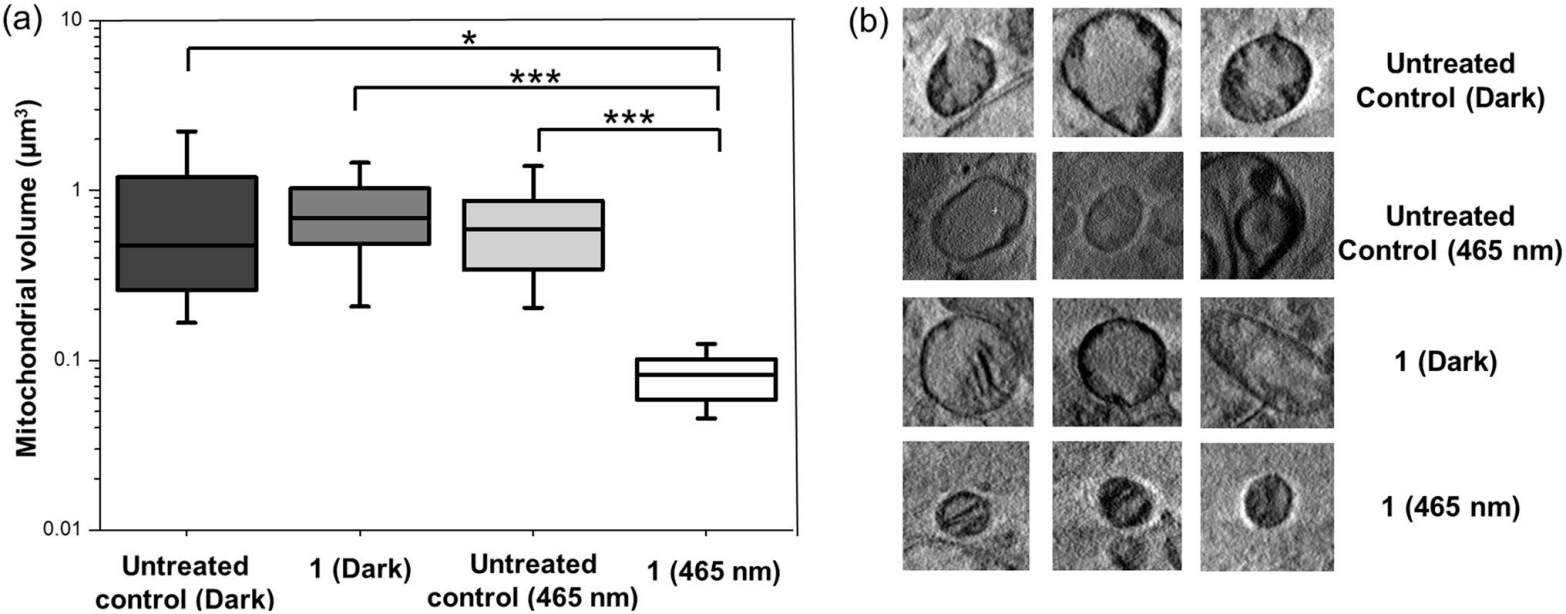
Changes in mitochondrial size and structure upon treatment of PC3 cells with **1** followed by irradiation with blue light. **a** Box and whisker plot showing average volumes (μm^3^) of segmented mitochondria from cryogenically fixed PC3 prostate carcinoma cells untreated or treated with 1 μM of **1** for 2 h, exposed to 10 min of dark or blue light (465 nm), and followed by 24 h recovery in complex-free medium (37 °C, 5% CO_2_). Statistical analysis was performed using Welch’s unpaired *t* test (assuming unequal variables; *p < 0.05, **p < 0.01, ***p < 0.001). **b** Representative 2D 4 × 4 μm^2^ sections of X-ray tomograms showing mitochondria from cryogenically fixed PC3 prostate carcinoma cells untreated or treated with 1 μM of **1** for 2 h, exposed to 10 min of darkness or blue light (465 nm), and followed by 24 h recovery in complex-free media (37 °C, 5% CO_2_). Images were generated using OriginPro 2018 (**a**) [50] and IMOD software (**b**) [33, 34]

On the contrary, heavily altered smaller mitochondria, and severe membrane blebbing, indicating initiation of a cell-death process [48], were visible when cells were treated with 1 μM **1** and irradiated with blue light (Figs. 2b and 3d, Fig. S12–S14; Videos SV8–SV10). Segmentation of tomograms of the different samples using SuRVoS [35] allowed further morphological analysis. 3D volumes of the nuclei and a number of mitochondria from all samples were obtained, and results were visualised using the Amira 3D imaging software (Fig. 2d, Fig S15–S18; Videos SV11–SV14) [36]. The mitochondria in cells subjected to treatment with organo-iridium complex **1** and irradiation were found to be a homogeneous population of small spherical organelles (0.08 ± 0.03 μm^3^, *N* = 19; range of volumes: 0.05–0.12 μm^3^; Fig. 4, Fig. S18, Table S1; Video SV14). In contrast, untreated cells (both non-irradiated and irradiated) and cells treated with **1**, but not exposed to blue light, contained larger and elongated mitochondria (untreated non-irradiated: 0.98 ± 1.2 μm^3^, *N* = 14; untreated irradiated: 0.65 ± 0.35 μm^3^, *N* = 20; 1 μM **1** non-irradiated: 0.77 ± 0.38 μm^3^, *N* = 26; Fig. 4, Fig. S15–S17, Table S1; Videos SV11–SV13), which were similar to organelles found normally in healthy cells (size from 0.75–3 μm^2^ in length) [49].

The alterations observed in mitochondria of PC3 cells treated with **1** and irradiated must be caused by ^1^O_2_ generated by complex located within the proximity of the organelle, as singlet oxygen (^1^O_2_) is short lived (< 200 ns in vitro) [51, 52], possesses a low capacity to diffuse within the cell (up to 1 µm) [53–55], and is not accumulated inside them. Previous experiments using optical microscopy had shown that **1** was broadly distributed within the cytosol of cells [1]. Moreover, detailed analysis of mitochondria from non-irradiated cells treated similarly with **1** showed the presence of low-electron-density (light) areas in their interior, surrounded by darker external membranes and cristae (Figs. 3, 4). Ir accumulation within the mitochondria would make it harder for soft X-rays to penetrate, turning them darker in appearance. Therefore, it is unlikely that the complex is able to penetrate to the interior of mitochondria under normal circumstances. Thus, the dark nature of mitochondria found in cells treated with **1** and irradiated might be due to an overall reduction in their size (condensing the outer membrane and internal cristae into a smaller volume).

## Conclusions

We have used synchrotron soft X-ray tomography to study the effects of visible-light photoactivation of the organo iridium photosensitiser **1** on PC3 human prostate cancer cells. The cells were cryopreserved after treatment to maintain them as close to their native state as possible. Dramatic morphological differences were observed between treated-irradiated (activated **1** at 1/6th IC_50_) and control cells. These included apparent mitochondrial alterations, most likely induced by singlet oxygen generated upon irradiation of Ir complex within the proximity of these organelles. It is possible that increases in the levels of a number of enzymes in the glycolytic pathway observed previously upon treatment of cells with **1** followed by irradiation with blue light [1] might be linked to changes in mitochondrial physiology, although this requires further investigation. Overall, we have demonstrated that cryo-SXT can provide direct insights into the effects of PDT agents on organelles in cancer cells. Such experiments will enhance the development of new PDT photosensitisers, and accelerate their progression towards more advanced stages of clinical development.

## Electronic supplementary material

Below is the link to the electronic supplementary material.

Supplementary file1 (DOCX 4610 kb)

Video. SV1 Video of a reconstructed X-ray tomogram (16 x 16 μm^2^) showing cellular and organelle morphology of a cryo-preserved PC3 human prostate carcinoma cell grown on carbon-gold TEM grid under dark conditions (not irradiated with blue light; 465 nm) followed by 24 h recovery in complex-free mediun (37 °C, 5% CO_2_; Tomogram 1, T1). (AVI 180403 kb)

Video. SV2 Video of a reconstructed X-ray tomogram (16 x 16 μm^2^) showing cellular and organelle morphology of a cryo-preserved PC3 human prostate carcinoma cell grown on carbon-gold TEM grid under dark conditions (not irradiated with blue light; 465 nm) followed by 24 h recovery in complex-free mediun (37 ºC, 5% CO_2_; Tomogram 2, T2). (AVI 143606 kb)

Video. SV3 Video of a reconstructed X-ray tomogram (16 x 16 μm^2^) showing cellular and organelle morphology of a cryo-preserved PC3 human prostate carcinoma cell grown on carbon-gold TEM grid and irradiated for 10 min at 465 nm followed by 24 h recovery in complex-free medium (37 ºC, 5% CO_2_; Tomogram 3, T3). (AVI 76612 kb)

Video. SV4 Video of a reconstructed X-ray tomogram (16 x 16 μm^2^) showing cellular and organelle morphology of a cryo-preserved PC3 human prostate carcinoma cell grown on carbon-gold TEM grid and treated with 1 μM of 1 for 2 h under dark conditions (not irradiated with blue light; 465 nm) followed by 24 h recovery in complex-free mediun (37 ºC, 5% CO_2_; Tomogram 4, T4).(AVI 105902 kb)

Video. SV5 Video of a reconstructed X-ray tomogram (16 x 16 μm^2^) showing cellular and organelle morphology of a cryo-preserved PC3 human prostate carcinoma cell grown on carbon-gold TEM grid and treated with 1 μM of 1 for 2 h under dark conditions (not irradiated with blue light; 465 nm) followed by 24 h recovery in complex-free mediun (37 ºC, 5% CO_2_; Tomogram 5, T5). (AVI 114452 kb)

Video. SV6 Video of a reconstructed X-ray tomogram (16 x 16 μm^2^) showing cellular and organelle morphology of a cryo-preserved PC3 human prostate carcinoma cell grown on carbon-gold TEM grid and treated with 1 μM of 1 for 2 h under dark conditions (not irradiated with blue light; 465 nm) followed by 24 h recovery in complex-free mediun (37 ºC, 5% CO_2_; Tomogram 6, T6). (AVI 102246 kb)

Video. SV7 Video of a reconstructed X-ray tomogram (16 x 16 μm^2^) showing cellular and organelle morphology of a cryo-preserved PC3 human prostate carcinoma cell grown on carbon-gold TEM grid and treated with 1 μM of 1 for 2 h under dark conditions (not irradiated with blue light; 465 nm) followed by 24 h recovery in complex-free mediun (37 ºC, 5% CO_2_; Tomogram 7, T7). (AVI 54311 kb)

Video. SV8 Video of a reconstructed X-ray tomogram (16 x 16 μm^2^) showing cellular and organelle morphology of a cryo-preserved PC3 human prostate carcinoma cell grown on carbon-gold TEM grid and treated with 1 μM of 1 for 2 h and irradiated for 10 min at 465 nm followed by 24 h recovery in complex-free medium (37 ºC, 5% CO_2_; Tomogram 8, T8). (AVI 128396 kb)

Video. SV9 Video of a reconstructed X-ray tomogram (16 x 16 μm^2^) showing cellular and organelle morphology of a cryo-preserved PC3 human prostate carcinoma cell grown on carbon-gold TEM grid and treated with 1 μM of 1 for 2 h and irradiated for 10 min at 465 nm followed by 24 h recovery in complex-free medium (37 ºC, 5% CO_2_; Tomogram 9, T9). (AVI 86329 kb)

Video. SV10 Video of a reconstructed X-ray tomogram (16 x 16 μm^2^) showing cellular and organelle morphology of a cryo-preserved PC3 human prostate carcinoma cell grown on carbon-gold TEM grid and treated with 1 μM of 1 for 2 h and irradiated for 10 min at 465 nm followed by 24 h recovery in complex-free medium (37 ºC, 5% CO_2_; Tomogram 10, T10). (AVI 96355 kb)

Video. SV11 Video of X-ray tomogram (Tomogram 1, T1) showing the volumes of mitochondria (orange) and the cell nucleus (green) of a cryogenically-fixed PC3 human prostate carcinoma cell grown on a carbon-gold TEM grid under dark conditions (not irradiated with blue light; 465 nm). A total of 14 mitochondria were identified and segmented. (MPG 53304 kb)

Video. SV12 Video of X-ray tomogram (Tomogram 3, T3) showing the volumes of mitochondria (orange) and the cell nucleus (green) of a cryogenically-fixed PC3 human prostate carcinoma cell grown on a carbon-gold TEM grid and exposed to 10 min irradiation with blue light (465 nm). A total of 20 mitochondria were identified and segmented. (MPG 85817 kb)

Video. SV13 Video of X-ray tomogram (Tomogram 4, T4) showing the volumes of mitochondria (orange) and the cell nucleus (green) of a cryogenically-fixed PC3 human prostate carcinoma cell grown on carbon-gold TEM grids, treated with 1 µM of 1 for 2 h under dark conditions (not irradiated with blue light; 465 nm). A total of 26 mitochondria were identified and segmented. (MPG 96321 kb)

Video. SV14 Video of X-ray tomogram (Tomogram 9, T9) showing the volumes of mitochondria (orange) and the cell nucleus (green) of a cryogenically-fixed PC3 human prostate carcinoma cell grown on a carbon-gold TEM grid, treated with 1 µM of 1 for 2 h followed by 10 min irradiation with blue light (465 nm) in complex-free medium. A total of 19 mitochondria were identified and segmented. (MPG 99248 kb)

## Abbreviations

AuNP: Gold nanoparticle fiducials
Cryo-SXT: Cryo-soft X-ray tomography
Cryo-TXM: Cryo-transmission X-ray microscope
DMSO: Dimethylsulfoxide
GFP: Green fluorescent protein
IC_50_: Half-maximal inhibitory concentration
PBS: Phosphate-buffered saline
PC3: Human prostate cancer cells
PDT: Photodynamic therapy
ROS: Reactive oxygen species
ROI: Region of interest
RPMI: Roswell Park Memorial Institute
SuRVoS: Super region volume segmentation
TEM: Transmission electron microscopy
