# Supplementary material for: X-ray tomography of cryopreserved human prostate cancer cells: mitochondrial targeting by an organoiridium photosensitiser
Source: J Biol Inorg Chem. 2020 Mar 2;25(2):295–303. doi: 10.1007/s00775-020-01761-8 (PMC7082392; doi:10.1007/s00775-020-01761-8)
Supplement: Supplementary file 1 — Supplementary file1 (DOCX 4610 kb) [file 775_2020_1761_MOESM1_ESM.docx]

Electronic Supplementary Material

**X-ray tomography of cryopreserved human prostate cancer cells: mitochondrial targeting by an organo-irodium photosensitiser**

Elizabeth M. Bolitho^a, b^, Carlos Sanchez-Cano^a c*^, Huaiyi Huang^a^, I. Hands-Portman^a^, Matthew Spink^b^, Paul D. Quinn^b^, Maria Harkiolaki^b*^, and Peter J. Sadler^a*^

*[a] Department of Chemistry and School of Life Sciences, Gibbett Hill Road, University of Warwick, Coventry, UK, CV4 7AL.University of Warwick, Coventry, UK, CV4 7AL.*

*[b] Diamond Light Source, OX11 0DE, Oxford, OX11 0DE*

*[c] CIC biomaGUNE, Paseo Miramon 182, 20014, San Sebastián, Gipuzkoa, Spain*

* Corresponding authors: [csanchez@cicbiomagune.es](mailto:csanchez@cicbiomagune.es)

[maria.harkiolaki@diamond.ac.uk](mailto:maria.harkiolaki@diamond.ac.uk)

[p.j.sadler@warwick.ac.uk](mailto:p.j.sadler@warwick.ac.uk)

**Supporting Information**

## **Materials and methods S1**

## **Instrumentation S2**

## **Cytotoxicity assays S3**

## **Table S1 S4**

## **Figures S1-S19 S5**

**Videos Tomograms SV1-SV10 Available on website**

**Videos segmented Tomograms SV11-SV14 Available on website**

## **Materials and Methods**

**Preparation of culture media.** RPMI-1640 medium (Scientific Laboratory Supplies, UK) was completed with FCS (10% v/v), Penicillin-Streptomycin mixture (1% v/v) and 2 mM glutamine solution (1% v/v), and the fully prepared medium solution were incubated at 37 °C before each use. RPMI-1460 no phenol-red was prepared in the same way. All stock solutions of **1** were prepared in 5% DMSO and 95% RPMI-1640 no phenol-red media.

**Defrosting cells.** Vials of frozen cells (containing 1 x 10^6^ cells per vial) from liquid nitrogen storage, were rapidly thawed in a 37 ºC water bath. The cell solution was then transferred to a 15 mL centrifuge tube (Scientific Laboratory Supplies, UK) and 4 mL of RPMI-1640 fully prepared medium were added and pipetted to ensure single cell suspension and even cell distribution. Cells were pelleted (22 ºC, 5 min, 1000 rpm) and the supernatant removed. The cell pellet was re-suspended in 3 mL of RPMI-1640 and transferred to a T25 cell culture flask (Greiner Bio-One, UK) for incubation and growth (37 ºC, 5% CO_2_).

**Passaging cells.** Once the cells had grown to ca. 80-90% coverage on the surface of the T25 (25 cm^2^) cell culture flask, the supernatant was removed and the cells washed with phosphate-buffered saline (PBS). PBS media were then aspirated and 0.25% trypsin/EDTA (1 mL) were added to cover the surface of the flask and left for 5 min (37 C, 5% CO2) to detach cells. RPMI-1640 (containing 10% FCS) was added to quench the trypsin activity, and pipetted multiple times to ensure a single cell suspension which was then transferred to a new T75 (75 cm^2^) culture flask. This process was repeated upon reaching 80-90% cell surface confluency, by typically passaging 1:4 (cell suspension: fresh media) and incubating at 37 C, under 5% CO_2_ humidified atmosphere for optimal cell growth.

## **Instrumentation**

**Plunge-freezer.** Plunge-freezing of TEM grids was performed on an in-house built manual plunge-freezer (School of Life Sciences, University of Warwick, UK) using a propane:ethane mixture (1:2) liquefied using liquid nitrogen.

**Microscopy imaging.** Preliminary brightfield, red-fluorescence and green-fluorescence mapping of samples was done on a Zeiss Axioimager with Linkam cryostage for grid preliminary analysis and mapping (max resolution 50x, variable filters) at the B24 beamline (Diamond Light Source, UK)

A grid pattern can be observed in some of the Mitotracker Red images shown below (but not in all). This pattern is caused by the mesh from the gold TEM grid where the cells were grown, and is due to the orientation in which grids were placed when mapped on the cryostage. Handling of the grids under low temperature conditions is complicated and the main aim of the mapping was to identify cells suitable for cryo-SXT. Therefore, we did not risk damage to the grids by changing the orientation for microscopy.

**Cytotoxicity assays**

**Sulforhodamine (SRB) assay.** A cell suspension of 10,000 PC3 cells per 200 μL was prepared in RMPI-1640 culture medium and seeded into black-out 96-well plates for 24 h (37 °C, 5% CO_2_). The cells were treated with 0.01- 100 µM of **1** (prepared in 5% DMSO and 95% RPMI-1640 without phenol red) for 2 h. The supernatant was removed, cells washed with PBS and complex-free RPMI-1640 non phenol-red medium (200 μL) was added to each well. Cells were then exposed to 10 min irradiation (465 nm, 4.8 mw/cm^2^), or left in the dark, before incubating for 46 h (37 °C, 5% CO_2_). After 48 h (2 h complex + 46 h recovery), cold 50% trichloroacetic acid (50 μL, Sigma-Aldrich, UK) was added to each well and kept at 4 C for 1 h. The plates were washed with distilled water (x7), dried and sulforhodamine B (SRB) dye (50 μL, Sigma-Aldrich, UK) was added to each well for 30 min. Plates were washed with 1% acetic acid (x7), dried thoroughly (hairdryer) and 1 M tris-base (150 μL) was added to each well for 1 h, and then analysed on a Bio-Rad MultiSkan plate reader at 492 and 680 nm. Data analysis was performed with Microsoft Excel and OriginPro 2016 software.

## **References**

[1] Luengo I, Darrow MC, Spink MC, Sun Y, Dai W, He CY, Chiu W, Pridmore T, Ashton AW, Duke EMH, Basham M, French AP (2017) SuRVoS: Super-Region Volume Segmentation workbench. J Struct Biol 198 (1):43-53. doi:10.1016/j.jsb.2017.02.007

[2] Rueden CT, Schindelin J, Hiner MC, DeZonia BE, Walter AE, Arena ET, Eliceiri KW (2017) ImageJ2: ImageJ for the next generation of scientific image data. BMC Bioinformatics 18 (1):529-529. doi:10.1186/s12859-017-1934-z

[3] Mastronarde DN, Held SR (2017) Automated tilt series alignment and tomographic reconstruction in IMOD. J Struct Biol 197 (2):102-113. doi:10.1016/j.jsb.2016.07.011

[4] Kremer JR, Mastronarde DN, McIntosh JR (1996) Computer visualization of three-dimensional image data using IMOD. Journal of structural biology 116 (1):71-76. doi:10.1006/jsbi.1996.0013

[5] Mittag U, Kriechbaumer A, Rittweger J (2017) A novel interpolation approach for the generation of 3D-geometric digital bone models from image stacks. J Musculoskelet Neuronal Interact 17 (2):86-96

**Table S1**

**Table S1.** The average calculated volumes (µm^3^) of individually segmented mitochondria in cryogenically-fixed PC3 cells gown on carbon-gold TEM grids, untreated or treated with 1 μM **1** under dark or irradiated (λ = 465 nm) conditions as estimated using SuRVoS through the generation of supervoxels.^[1]^ A total of 4 representative tomograms (one for each condition) have been volume segmented and labelled as per the tomograms in **figures S5**-**14 (T1-10)**: (i) untreated control (dark) = **T1**; (ii) untreated control (465 nm) = **T3**; (iii) treated (dark) = **T4**; (iv) treated (465 nm) = **T9**.

| Mitochondrion number | 3D volume (µm^3^) | | | |
| --- | --- | --- | --- | --- |
|  | **Untreated control (Dark)^[a]^** | **Untreated control (λ =** **465 nm)^[b]^** | **Treated (Dark)^[c]^** | **Treated**  **(λ =** **465 nm)^[d]^** |
| 1 | 1.193 | 0.785 | 1.444 | 0.0693 |
| 2 | 0.725 | 0.638 | 1.454 | 0.0498 |
| 3 | 0.401 | 0.952 | 1.001 | 0.0588 |
| 4 | 2.221 | 1.393 | 0.864 | 0.1010 |
| 5 | 4.405 | 1.132 | 0.733 | 0.1249 |
| 6 | 0.252 | 0.481 | 1.283 | 0.1211 |
| 7 | 0.553 | 0.568 | 0.384 | 0.0922 |
| 8 | 0.258 | 0.368 | 1.217 | 0.0739 |
| 9 | 0.167 | 0.619 | 0.564 | 0.0455 |
| 10 | 0.945 | 0.494 | 0.236 | 0.1030 |
| 11 | 0.228 | 1.293 | 0.223 | 0.0558 |
| 12 | 0.361 | 0.638 | 0.914 | 0.1197 |
| 13 | 1.650 | 0.304 | 1.348 | 0.0463 |
| 14 | 0.312 | 0.204 | 0.609 | 0.0898 |
| 15 |  | 0.292 | 0.410 | 0.0615 |
| 16 |  | 1.019 | 0.722 | 0.0991 |
| 17 |  | 0.562 | 1.197 | 0.0942 |
| 18 |  | 0.702 | 0.602 | 0.0693 |
| 19 |  | 0.313 | 1.026 | 0.0693 |
| 20 |  | 0.315 | 0.511 |  |
| 21 |  |  | 0.479 |  |
| 22 |  |  | 0.569 |  |
| 23 |  |  | 0.207 |  |
| 24 |  |  | 0.487 |  |
| 25 |  |  | 0.748 |  |
| 26 |  |  | 0.656 |  |
| Mean | **0.98 ± 1.2** | **0.65 ± 0.35** | **0.77 ± 0.38** | **0.08 ± 0.03** |

^[a]^Segmented mitochondrial organelles in PC3 cells grown on carbon-gold TEM grids and exposed to dark conditions for 2 h followed by 24 h recovery in complex-free media, as calculated from **tomogram 1** (**T1**). ^[b]^Segmented mitochondrial organelles in PC3 cells grown on carbon-gold TEM grids for 2 h, followed by 10 min irradiation (λ = 465 nm) and 24 h recovery in complex-free media, as calculated from **tomogram 3 (T3).** ^[c]^Segmented mitochondrial organelles in PC3 cells grown on carbon-gold TEM grids and treated with **1** (1 μM) for 2 h under dark conditions followed by 24 h recovery in complex-free media, as calculated from **tomogram 4 (T4).** ^[d]^Segmented mitochondrial organelles in PC3 cells grown on carbon-gold TEM grids and treated with **1** (1 μM) for 2 h followed by 10 min irradiation (λ = 465 nm) and 24 h recovery in complex-free media (37 C, 5% CO_2_), as calculated from **tomogram 9 (T7).** Statistical analysis was performed using Welch’s unpaired t-test (assuming non-equal variables) which revealed no statistically significant differences between mitochondrial volumes of the untreated controls (light and dark) and the cells treated with **1** in the dark ([a-c], p-values > 0.05), however, the mitochondrial sizes in cells treated with **1** and exposed to blue light ([d]) were highly statistically different compared to the irradiated untreated controls (p = 0.0001).

**Figures S1-S19**


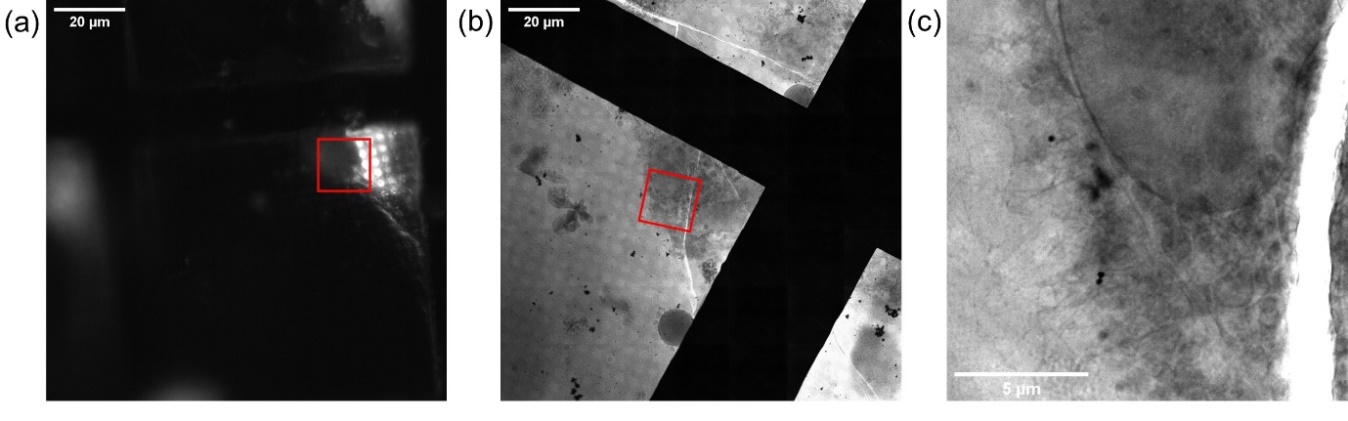


**Fig. S1** Cryo-SXT images of cryo-preserved untreated PC3 cells grown on carbon-gold TEM grids kept in the dark (not irradiated with blue light; 465 nm) followed by 24 h recovery in complex-free media and incubation with MitotrackerRed (λ_ex/em_ = 581/644 nm). (a) Red-fluorescent microscopy images taken on a Zeiss Axioimager coupled to a Linkam cryostage. (b) X-ray mosaic of the same area (100 x 100 μm^2^). (c) 2D X-ray projection (16 x 16 μm^2^) of the region of interest (indicated by red box). Differences in the orientation of the region of interest (red box) are due to variability in grid orientation upon sample transfer to different microscopes. Images were generated in ImageJ^[2]^  (a-b) and IMOD^[3,4]^ (c) imaging software.


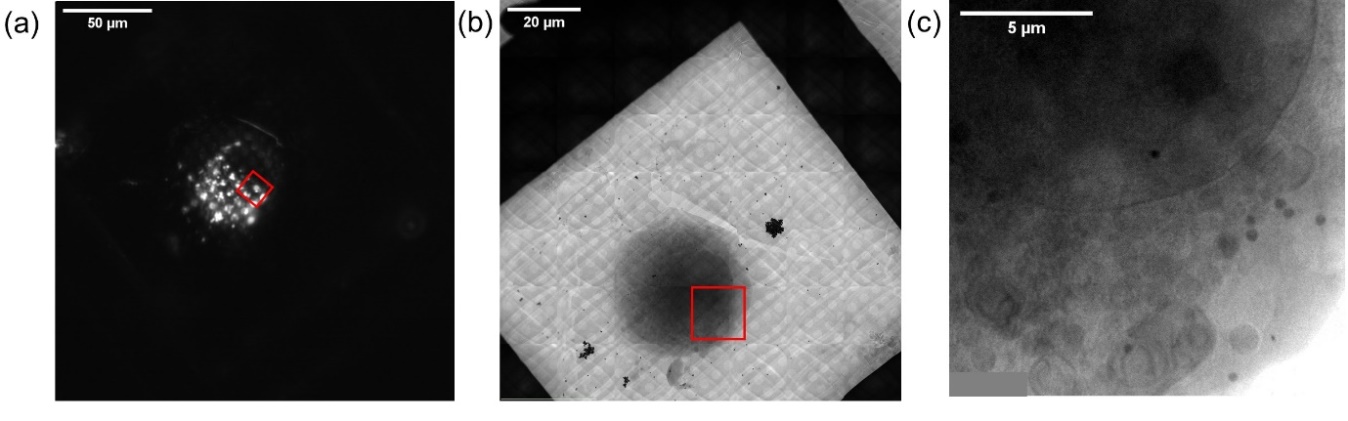


**Fig. S2** Cryo-SXT images of cryo-preserved untreated PC3 cells grown on carbon-gold TEM grids exposed to irradiation (10 min, 465 nm) followed by 24 h recovery in complex-free media and incubation with MitotrackerRed (λ_ex/em_ = 581/644 nm). (a) Red-fluorescent microscopy images taken on a Zeiss Axioimager coupled to a Linkam cryostage. (b) X-ray mosaic of the same area (100 x 100 μm^2^). (c) 2D X-ray projection (16 x 16 μm^2^) of the region of interest (indicated by red box). Differences in the orientation of the region of interest (red box) are due to variability in grid orientation upon sample transfer to different microscopes. Images were generated in ImageJ^[2]^  (a-b) and IMOD^[3,4]^ (c) imaging software.


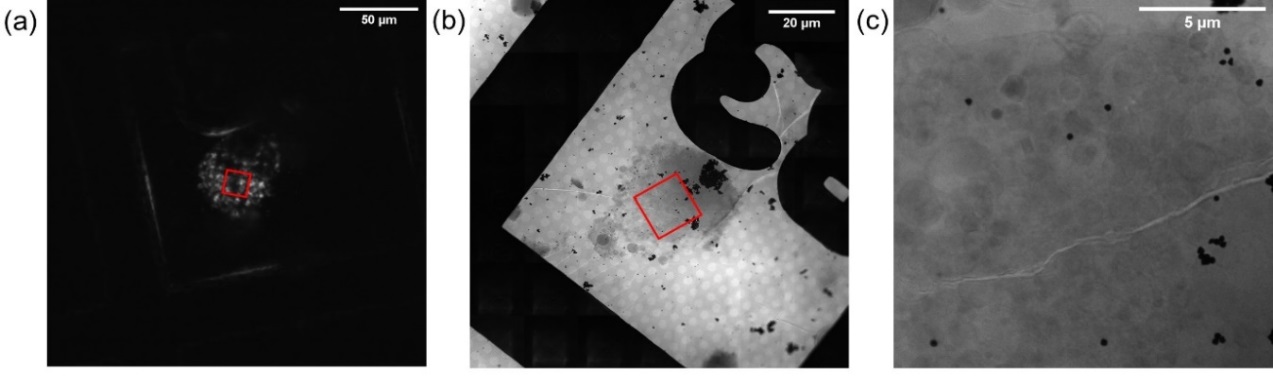


**Fig. S3** Cryo-SXT images of cryo-preserved PC3 cells grown on carbon-gold TEM grids treated with 1 μM **1** for 2 h and exposed to dark conditions (not irradiated with blue light; 465 nm) followed by 24 h recovery in complex-free media and incubation with MitotrackerRed (λ_ex/em_ = 581/644 nm). (a) Red-fluorescent microscopy images taken on Zeiss Axioimager coupled to a Linkam cryostage. (b) X-ray mosaic of the same area (100 x 100 μm^2^). (c) 2D X-ray projection (16 x 16 μm^2^) of the region of interest (indicated by red box). Ice crack observed in (c) is a common side effect of the blotting and plunge freezing process. Differences in the orientation of the region of interest (red box) are due to variability in grid orientation upon sample transfer to different microscopes. Images were generated in ImageJ^[2]^  (a-b) and IMOD^[3,4]^ (c) imaging software.


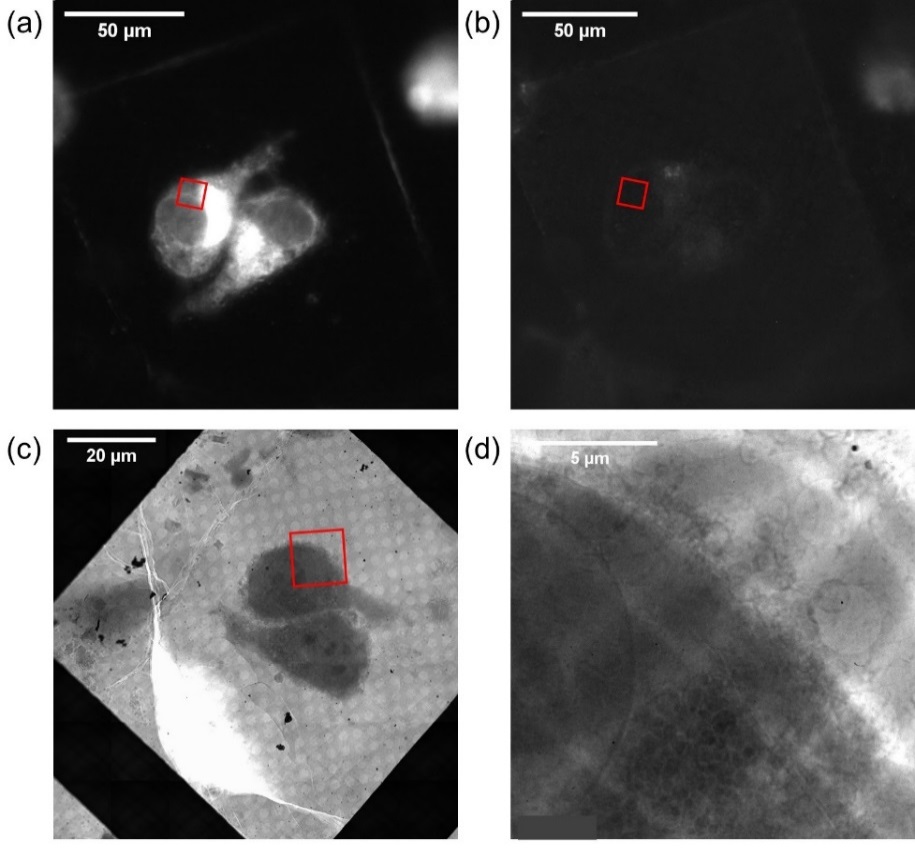


**Fig. S4** Cryo-SXT images of cryo-preserved PC3 cells grown on carbon-gold TEM grids treated with 1 μM **1** for 2 h and exposed to blue light (10 min, 465 nm) followed by 24 h recovery in complex-free media and incubation with MitotrackerRed (λ_ex/em_ = 581/644 nm­). (a) Red-fluorescent (λ_ex/em_ = 581/644 nm­) microscopy images taken on a Zeiss Axioimager coupled to a Linkam cryostage. (b) Green-fluorescent (λ_ex/em_ =458/596 nm) microscopy images taken on Zeiss Axioimager coupled to a Linkam cryostage. (c) X-ray mosaic (100 x 100 μm^2^). (d) 2D X-ray projection (16 x 16 μm^2^) of the region of interest (indicated by red box). The appearance of (d) varies to that of the tomograms observed in Fig S1-3 (pattern of light) as it was not background corrected. Differences in the orientation of the region of interest (red box) are due to variability in grid orientation upon sample transfer to different microscopes. Images were generated in ImageJ^[2]^  (a-b) and IMOD^[3,4]^ (c) imaging software.

**
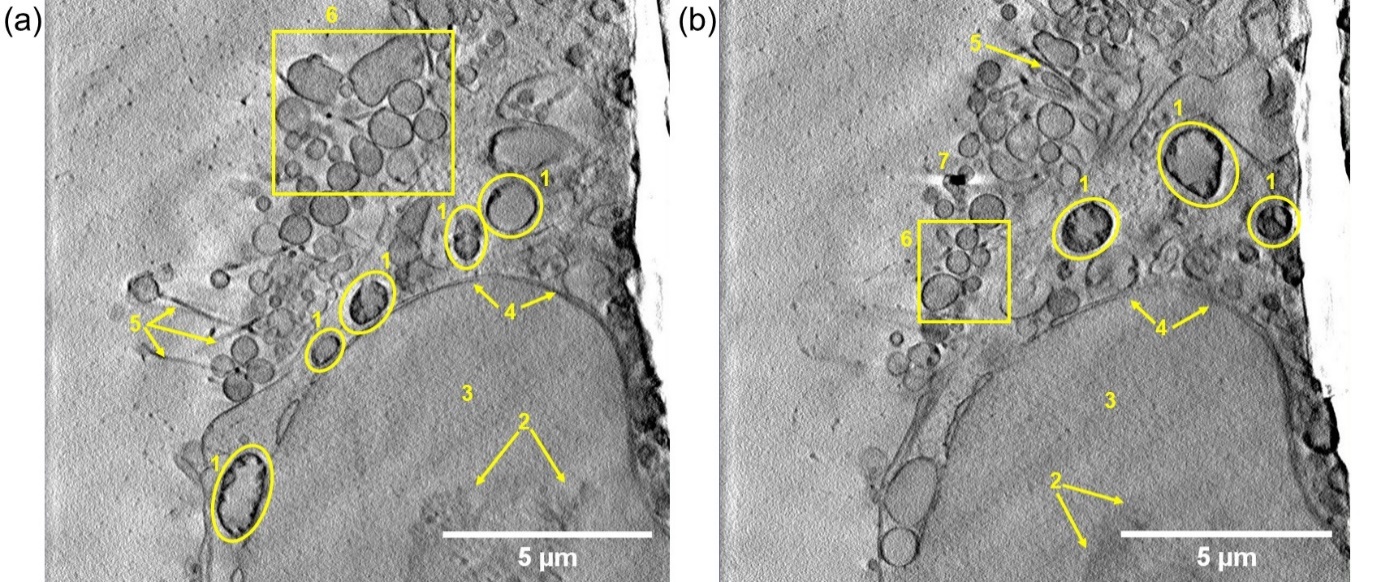
**

**Fig. S5** 2D projections of a reconstructed X-ray tomogram (16 x 16 μm^2^) showing cellular and organelle morphology of a cryo-preserved PC3 human prostate carcinoma cell grown on carbon-gold TEM grid under dark conditions (not irradiated with blue light; 465 nm) followed by 24 h recovery in complex-free mediun (37 C, 5% CO_2_; **Tomogram 1**, **T1**) visualized at two different sample tilt angles, obtained from the same area shown in Fig. S1: (a) 0°; (b) +28°, showing 1 = mitochondria, 2 = nucleolus, 3 = nucleus, 4 = nuclear membrane, 5 = features of lamellipodium, 6 = spherical vesicles and 7 = gold nanoparticle fiducials (d = 250 nm). Whole tomogram can be seen in Video SV1. Images were generated in IMOD^[3,4]^ (a-b) imaging software.

**
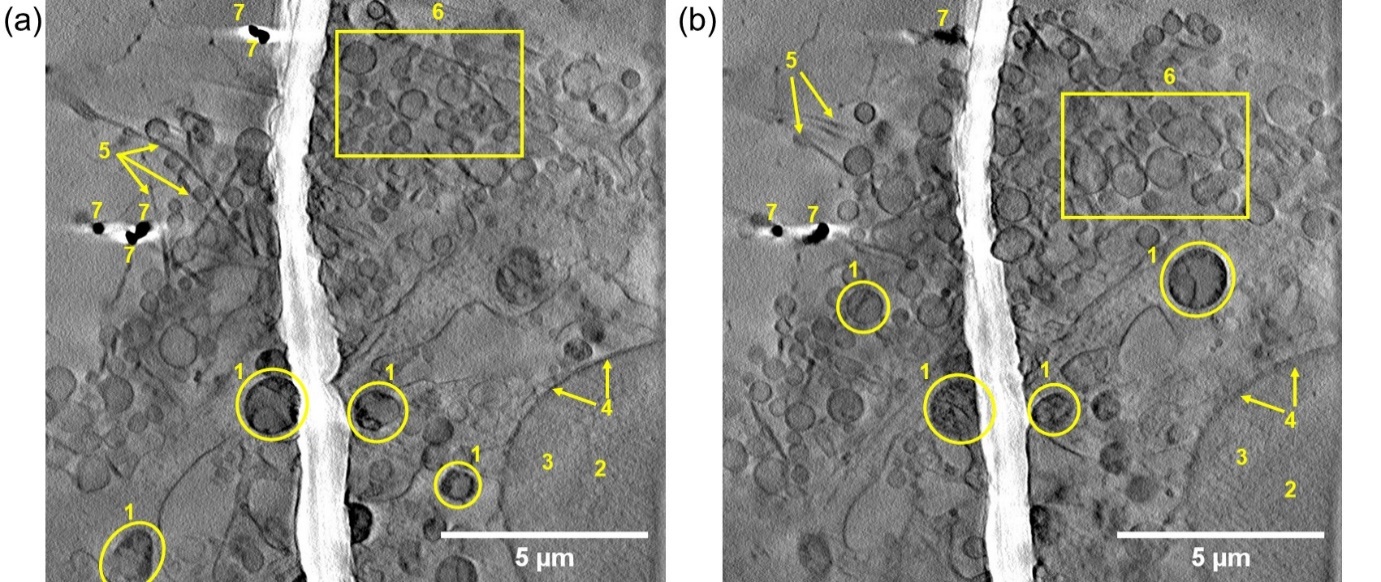
**

**Fig. S6** 2D projections of a reconstructed X-ray tomogram (16 x 16 μm^2^) showing cellular and organelle morphology of a cryo-preserved PC3 human prostate carcinoma cell grown on carbon-gold TEM grid under dark conditions (not irradiated with blue light; 465 nm) followed by 24 h recovery in complex-free mediun (37 C, 5% CO_2_; **Tomogram 2**, **T2**) at two different sample tilt angles: (a) 0°; (b) +14°, showing 1 = mitochondria, 2 = nucleolus, 3 = nucleus, 4 = nuclear membrane, 5 = features of lamellipodium, 6 = spherical vesicles and 7 = gold nanoparticle fiducials (d = 250 nm). Whole tomogram can be seen in Video SV2. Images were generated in IMOD^[3,4]^ (a-b) imaging software.

**
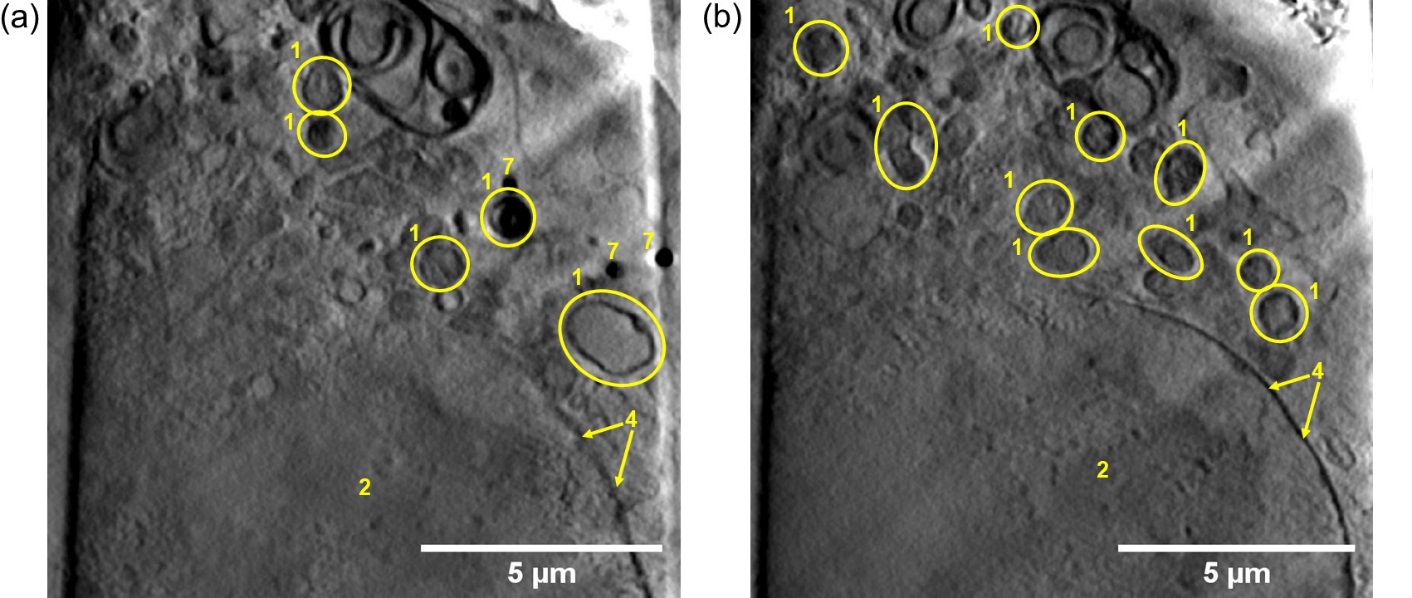
**

**Fig. S7** 2D projections of a reconstructed X-ray tomogram (16 x 16 μm^2^) showing cellular and organelle morphology of a cryo-preserved PC3 human prostate carcinoma cell grown on carbon-gold TEM grid and irradiated for 10 min at 465 nm followed by 24 h recovery in complex-free medium (37 C, 5% CO_2_; **Tomogram 3**, **T3**) at two different sample tilt angles obtained from the same area visulaized in Fig. S2.: (a) 0°; (b) +8°, showing 1 = mitochondria, 2 = nucleolus, 3 = nucleus, 4 = nuclear membrane, 5 = features of lamellipodium, 6 = spherical vesicles and 7 = gold nanoparticle fiducials (d = 250 nm). Whole tomogram can be seen in Video SV3. Images were generated in IMOD^[3,4]^ (a-b) imaging software.


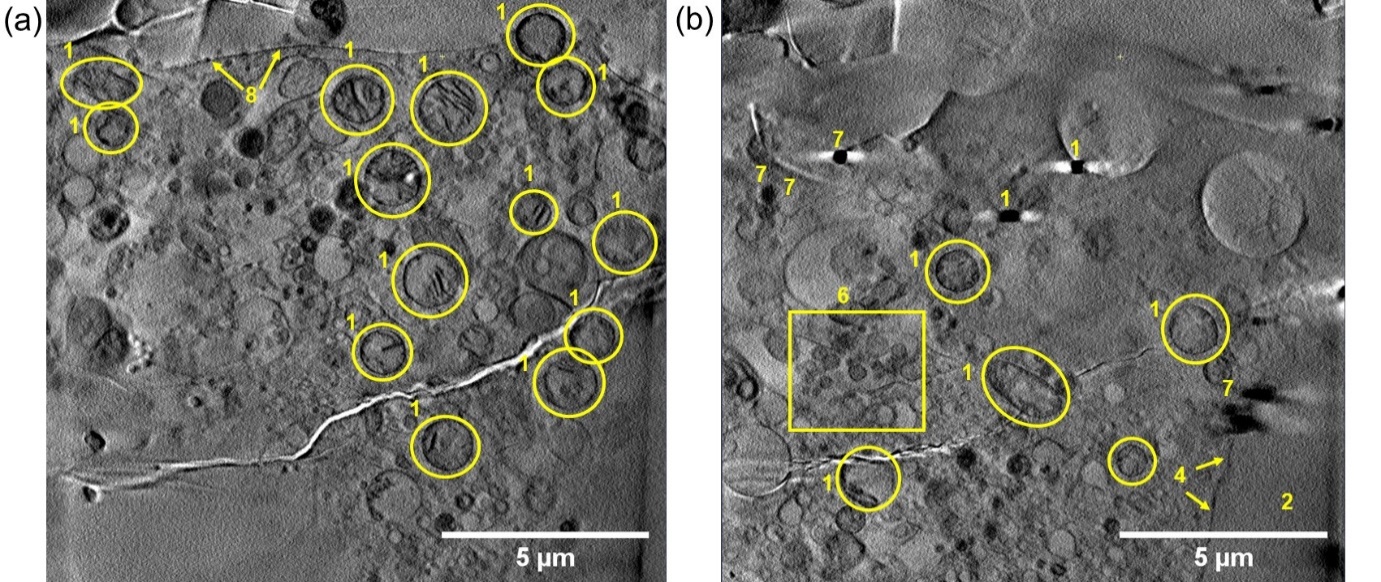


**Fig. S8** 2D projections of a reconstructed X-ray tomogram (16 x 16 μm^2^) showing cellular and organelle morphology of a cryo-preserved PC3 human prostate carcinoma cell grown on carbon-gold TEM grid and treated with 1 μM of **1** for 2 h under dark conditions (not irradiated with blue light; 465 nm) followed by 24 h recovery in complex-free mediun (37 C, 5% CO_2_; **Tomogram 4**, **T4**) at two sample tilt angles as obtained from the same area in Fig S3: (a) 0°; (b) +54°, showing 1 = mitochondria, 2 = nucleolus, 3 = nucleus, 4 = nuclear membrane, 5 = features of lamellipodium, 6 = spherical vesicles, 7 = gold nanoparticle fiducials (d = 250 nm) and 8 = plasma membrane. Whole tomogram can be seen in Video SV4. Images were generated in IMOD^[3,4]^ (a-b) imaging software.


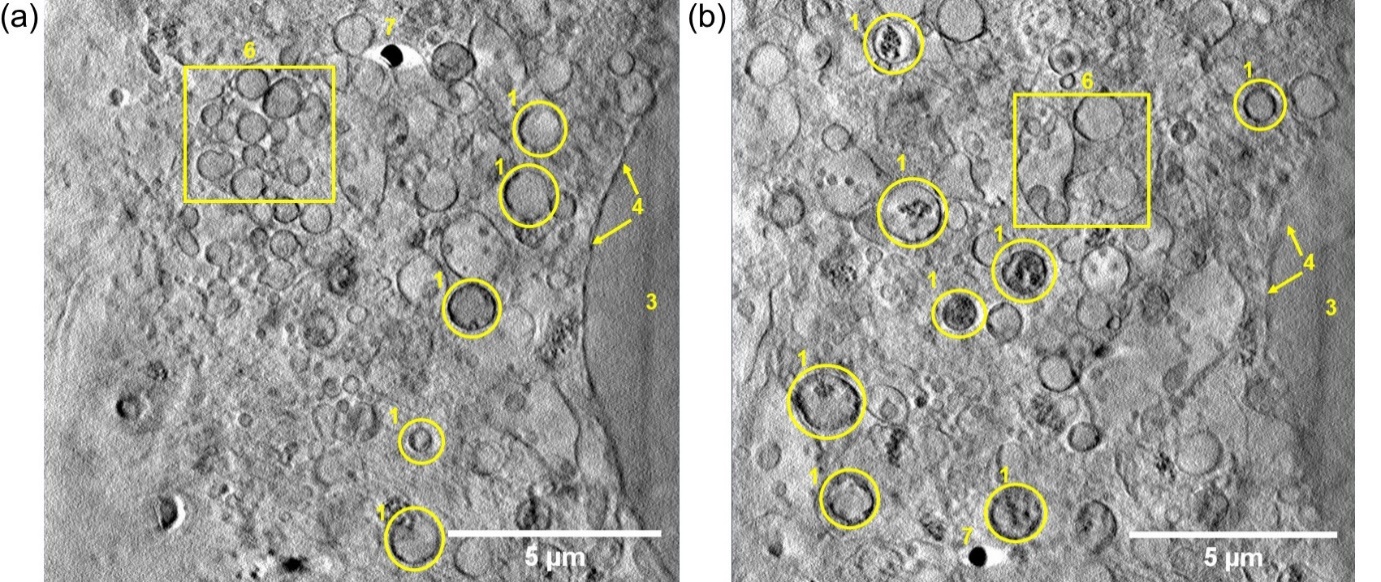


**Fig. S9** 2D projections of a reconstructed X-ray tomogram (16 x 16 μm^2^) showing cellular and organelle morphology of a cryo-preserved PC3 human prostate carcinoma cell grown on carbon-gold TEM grid and treated with 1 μM of **1** for 2 h under dark conditions (not irradiated with blue light; 465 nm) followed by 24 h recovery in complex-free medium (37 C, 5% CO_2_; **Tomogram 5**, **T5**) at two sample tilt angles: (a) 0°; (b) +15°, showing 1 = mitochondria, 2 = nucleolus, 3 = nucleus, 4 = nuclear membrane, 5 = features of lamellipodium, 6 = spherical vesicles and 7 = gold nanoparticle fiducials (d = 250 nm). Whole tomogram can be seen in Video SV5. Images were generated in IMOD^[3,4]^ (a-b) imaging software.


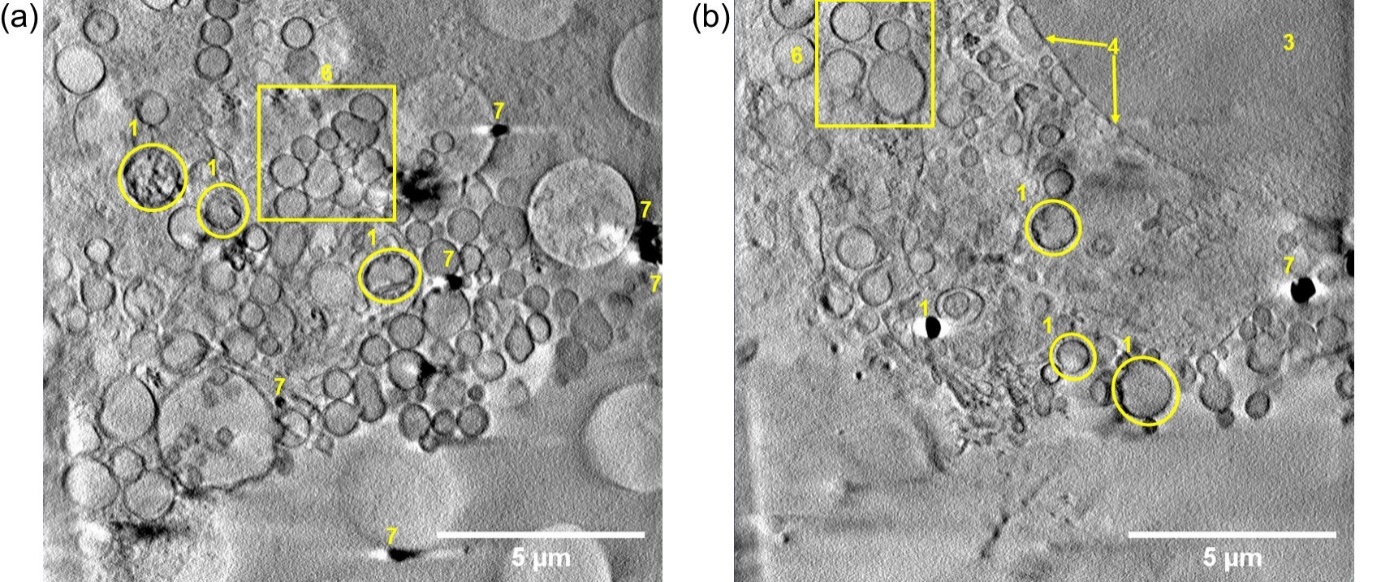


**Fig. S10** 2D projections of a reconstructed X-ray tomogram (16 x 16 μm^2^) showing cellular and organelle morphology of a cryo-preserved PC3 human prostate carcinoma cell grown on carbon-gold TEM grid and treated with 1 μM of **1** for 2 h under dark conditions (not irradiated with blue light; 465 nm) followed by 24 h recovery in complex-free medium (37 C, 5% CO_2_; **Tomogram 6**, **T6**) at two sample tilt angles: (a) 0°; (b) +24° showing 1 = mitochondria, 2 = nucleolus, 3 = nucleus, 4 = nuclear membrane, 5 = features of lamellipodium, 6 = spherical vesicles and 7 = gold nanoparticle fiducials (d = 250 nm). Whole tomogram can be seen in Video SV6. Images were generated in IMOD^[3,4]^ (a-b) imaging software.


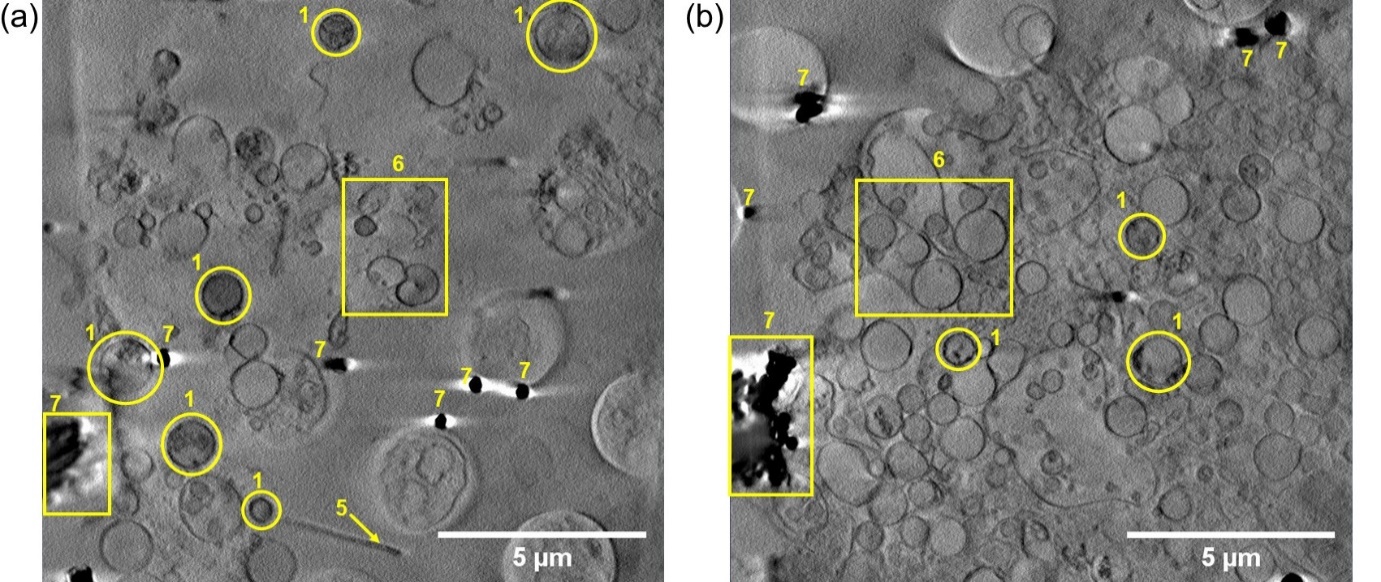


**Fig. S11** 2D projections of a reconstructed X-ray tomogram (16 x 16 μm^2^) showing cellular and organelle morphology of a cryo-preserved PC3 human prostate carcinoma cell grown on carbon-gold TEM grid and treated with 1 μM of **1** for 2 h under dark conditions (not irradiated with blue light; 465 nm) followed by 24 h recovery in complex-free medium (37 C, 5% CO_2_; **Tomogram 7**, **T7**) at two sample tilt angles: (a) 0°; (b) +39°, showing 1 = mitochondria, 2 = nucleolus, 3 = nucleus, 4 = nuclear membrane, 5 = features of lamellipodium, 6 = spherical vesicles and 7 = gold nanoparticle fiducials (d = 250 nm). Whole tomogram can be seen in Video SV7. Images were generated in IMOD^[3,4]^ (a-b) imaging software.


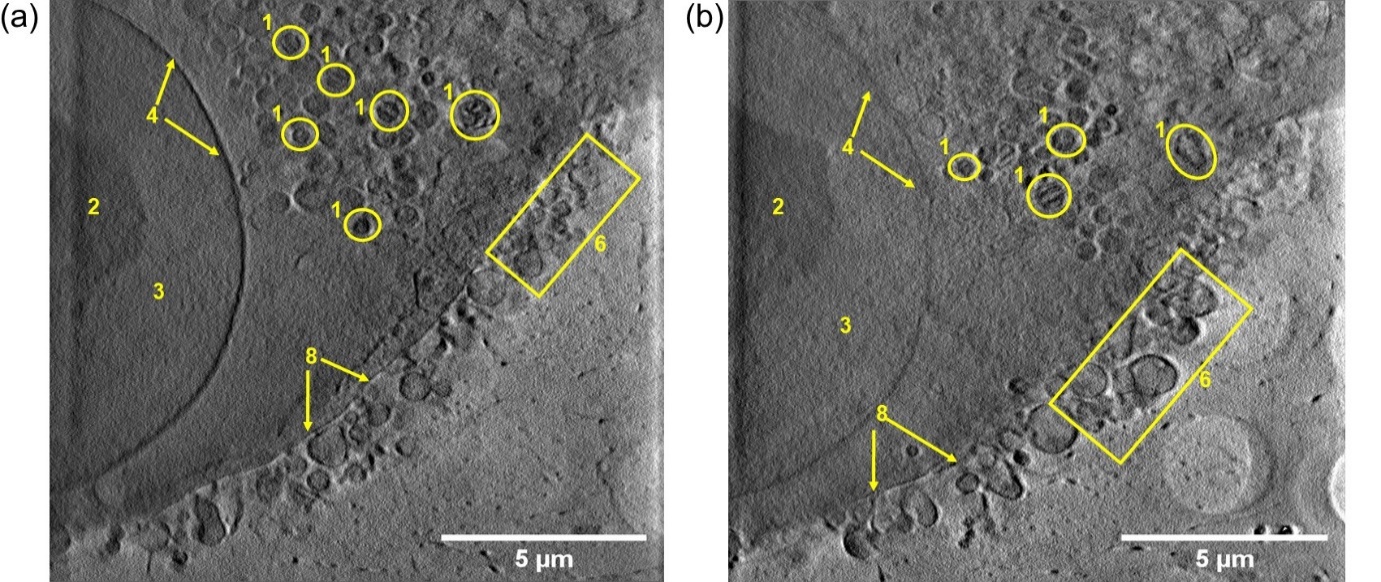


**Fig. S12** 2D projections of a reconstructed X-ray tomogram (16 x 16 μm^2^) showing cellular and organelle morphology of a cryo-preserved PC3 human prostate carcinoma cell grown on carbon-gold TEM grid and treated with 1 μM of **1** for 2 h and irradiated for 10 min at 465 nm followed by 24 h recovery in complex-free medium (37 C, 5% CO_2_; **Tomogram 8**, **T8**) at two sample tilt angles as obtained from the area shown in Fig.S4: (a) 0°; (b) -36°, showing 1 = mitochondria, 2 = nucleolus, 3 = nucleus, 4 = nuclear membrane, 5 = features of lamellipodium, 6 = spherical vesicles, 7 = gold nanoparticle fiducials (d = 250 nm) and 8 = plasma membrane. Whole tomogram can be seen in Video SV8. Images were generated in IMOD^[3,4]^ (a-b) imaging software.


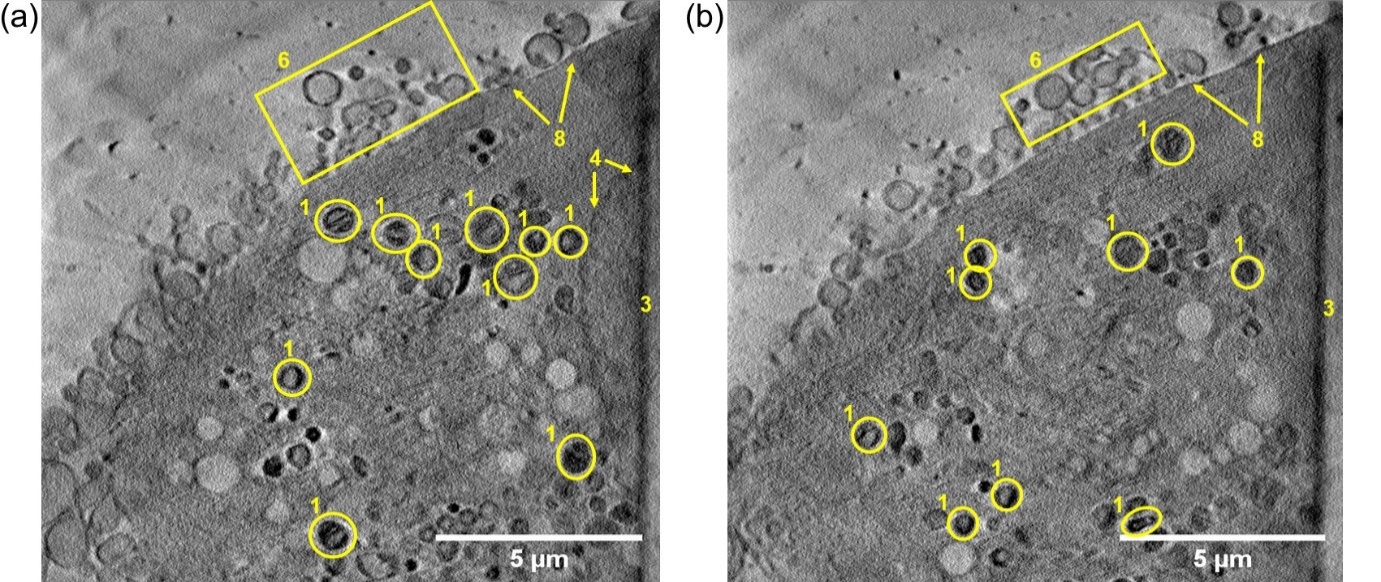


**Fig. S13** 2D projections of a reconstructed X-ray tomogram (16 x 16 μm^2^) showing cellular and organelle morphology of a cryo-preserved PC3 human prostate carcinoma cell grown on carbon-gold TEM grid and treated with 1 μM of **1** for 2 h, irradiated for 10 min at 465 nm followed by 24 h recovery in complex-free medium (37 C, 5% CO_2_;**Tomogram 9**, **T9**) at two sample tilt angles: (a) 0°; (b) +10°, showing 1 = mitochondria, 2 = nucleolus, 3 = nucleus, 4 = nuclear membrane, 5 = features of lamellipodium, 6 = spherical vesicles, 7 = gold nanoparticle fiducials (d = 250 nm) and 8 = plasma membrane. Whole tomogram can be seen in Video SV9. Images were generated in IMOD^[3,4]^ (a-b) imaging software.


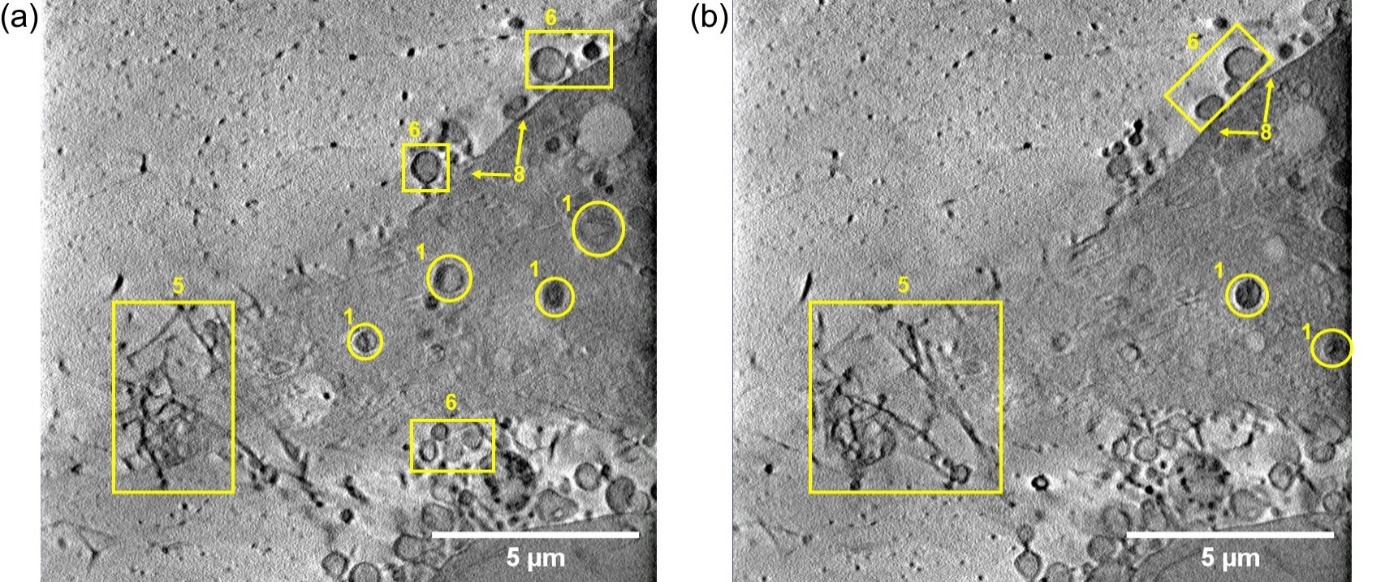


**Fig S14** 2D projections of a reconstructed X-ray tomogram (16 x 16 μm^2^) showing cellular and organelle morphology of a cryo-preserved PC3 human prostate carcinoma cell grown on carbon-gold TEM grid and treated with 1 μM of **1** for 2 h and irradiated for 10 min at 465 nm, followed by 24 h recovery in complex-free medium (37 C, 5% CO_2_;**Tomogram 10**, **T10**) at two sample tilt angles: (a) 0°; (b) +6.5°, showing 1 = mitochondria, 2 = nucleolus, 3 = nucleus, 4 = nuclear membrane, 5 = features of lamellipodium, 6 = spherical vesicles, 7 = gold nanoparticle fiducials (d = 250 nm) and 8 = plasma membrane. Whole tomogram can be seen in Video SV10. Images were generated in IMOD^[3,4]^ (a-b) imaging software.


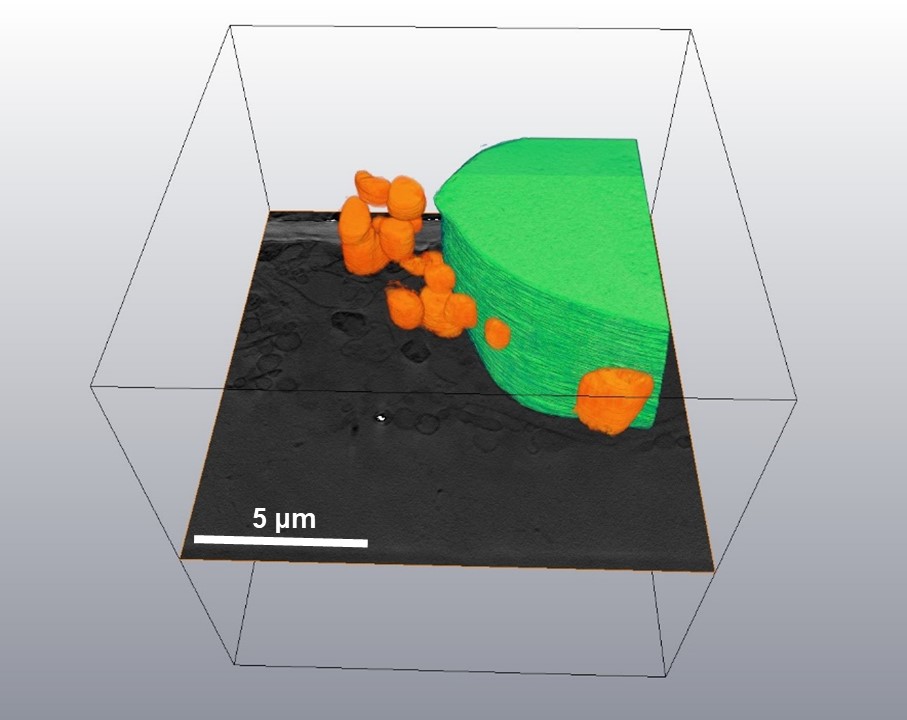


**Fig. S15** 3D projection of a segmented X-ray tomogram (**Tomogram 1**, **T1**) showing the volumes of mitochondria (orange) and the cell nucleus (green) of a cryogenically-fixed PC3 human prostate carcinoma cell grown on a carbon-gold TEM grid under dark conditions (not irradiated with blue light; 465 nm). A total of 14 mitochondria were identified and segmented. Whole sectioned tomogram can be seen in Video SV11. Images were generated using the Survos^[1]^ and Amira^[5]^ software.


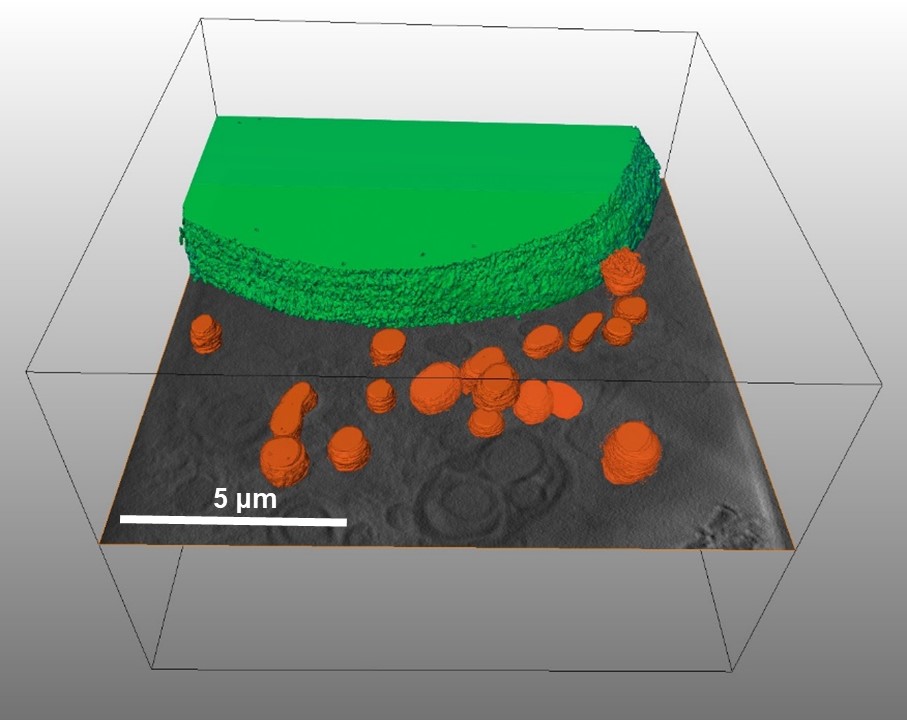


**Fig. S16** 3D projection of a segmented X-ray tomogram (**Tomogram 3**, **T3**) showing the volumes of mitochondria (orange) and the cell nucleus (green) of a cryogenically-fixed PC3 human prostate carcinoma cell grown on a carbon-gold TEM grid and exposed to 10 min irradiation with blue light (465 nm). A total of 20 mitochondria were identified and segmented. Whole sectioned tomogram can be seen in Video SV12. Images were generated using the Survos^[1]^ and Amira^[5]^ software.


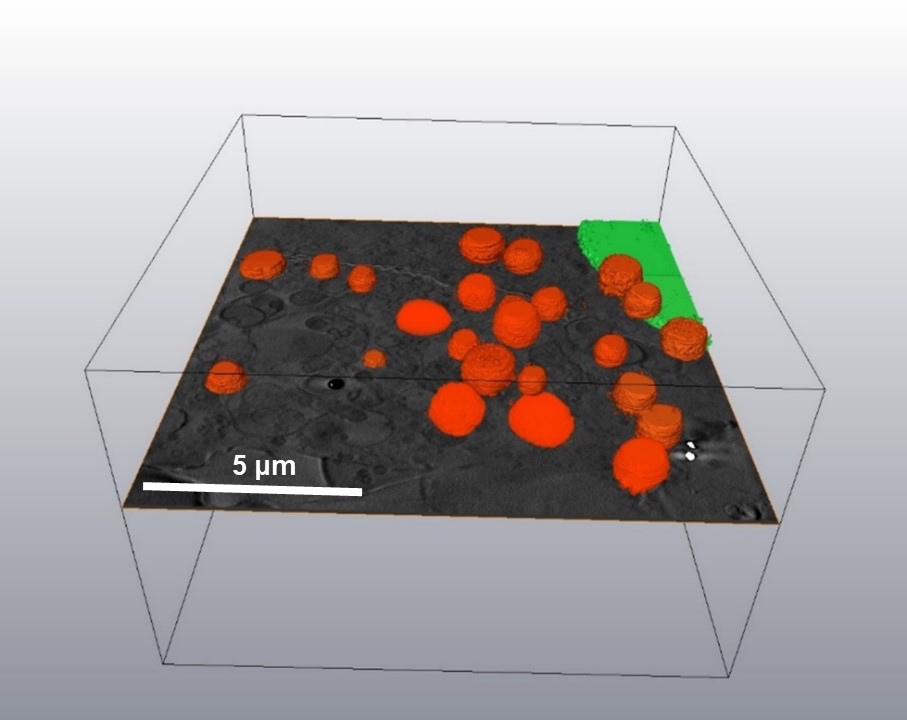


**Fig. S17** 3D projection of a segmented X-ray tomogram (**Tomogram 4**, **T4**) showing the volumes of mitochondria (orange) and the cell nucleus (green) of a cryogenically-fixed PC3 human prostate carcinoma cell grown on carbon-gold TEM grids, treated with 1 µM of **1** for 2 h under dark conditions (not irradiated with blue light; 465 nm). A total of 26 mitochondria were identified and segmented. Whole sectioned tomogram can be seen in Video SV13. Images were generated using the Survos^[1]^ and Amira^[5]^ software.


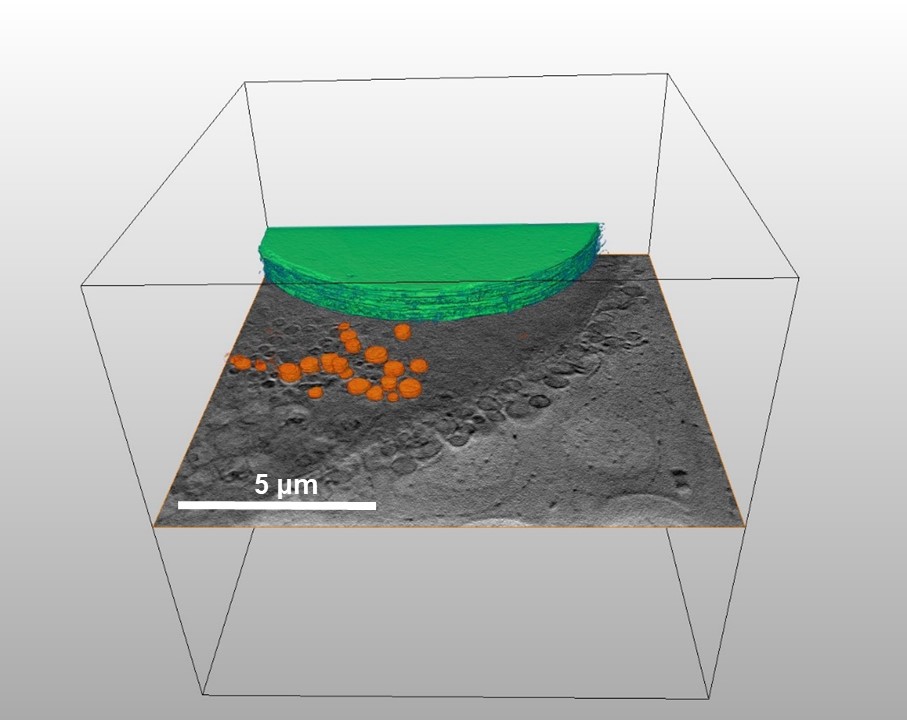


**Fig. S18** 3D projection of a segmented X-ray tomogram (**Tomogram 9**, **T9**) showing the volumes of mitochondria (orange) and the cell nucleus (green) of a cryogenically-fixed PC3 human prostate carcinoma cell grown on a carbon-gold TEM grid, treated with 1 µM of **1** for 2 h followed by 10 min irradiation with blue light (465 nm) in complex-free medium. A total of 19 mitochondria were identified and segmented. Whole sectioned tomogram can be seen in Video SV14. Images were generated using the Survos^[1]^ and Amira^[5]^ software.
